# Supplementary material for: Novel device for assisted vaginal birth: using integrated qualitative case study methodology to optimise Odon Device use within a feasibility study in a maternity unit in the Southwest of England
Source: BMJ Open. 2022 Aug 4;12(8):e059115. doi: 10.1136/bmjopen-2021-059115 (PMC9358957; doi:10.1136/bmjopen-2021-059115)
Supplement: Supplementary data [file bmjopen-2021-059115supp001.pdf]

REVISION RECORD		
REV (VER)	REVISION DESCRIPTION	CR NO.
01(A)	INITIAL RELEASE	CM500000111539

BD Odon Device™

Inflatable device for assisted vaginal birth – Instructions For Use

Device Description

The **BD Odon Device™** is an instrument for assisted vaginal birth. The device uses a polyethylene sleeve and an inflatable air cuff to help ease the fetal head out of the birth canal. The BD Odon Device consists of an applicator, sleeve and fastening band.

Indications for Use

The BD Odon Device is envisaged to perform assisted vaginal birth. The main indications for the BD Odon Device are as follows:

- Prolonged second stage of labour
- Suspected fetal compromise in the second stage of labour
- Other medical indications to shorten second stage of labour

Contraindications

The use of the BD Odon Device is contraindicated in the following conditions:

- Cervix is not fully dilated
- Cephalopelvic disproportion/ irreducible moulding and large caput indicative of cephalopelvic disproportion
- Fetal head not fully engaged
- Undefined presentation
- Breech, transverse, face, brow presentations
- Membranes are not ruptured
- Contraindications for vaginal deliveries
- Untrained operator

General Warnings

1. The BD Odon Device should be used by skilled birth attendants who are:
   a. Trained to recognize the conditions for safe and effective application of the BD Odon Device:
      - Full cervical dilation
      - Fetal head position defined
      - Fetal head station defined

   b. Trained to use the BD Odon Device
2. The BD Odon Device is intended for single use. Do not reuse. Device function may be impaired through reuse or cleaning. Degradation of materials or failure to correctly reassemble the device could result in harm for the mother and the fetus. Reusing the product after exposure to biological materials may cause adverse patient reactions due to contamination.
3. As with other devices for assisted vaginal birth, the BD Odon Device could fail to deliver the baby.
   The criteria to define failure and to abandon the procedure are:
   a. No progression in descent of the fetal head after a total of three pulls
   b. No more than two attempts at application (no more than two device slippages)
   c. Duration of procedure longer than 20 minutes
4. As with other instruments for assisted vaginal birth, use of the BD Odon Device may result in maternal and neonatal complications. The most common complications with the use of forceps and ventouse include maternal trauma, neonatal facial injury (forceps), cephalohaematoma, subgaleal haemorrhage, and retinal haemorrhage (ventouse).
   Presently human, animal and simulated studies have not identified potential complications specific to the BD Odon Device therefore this section will be updated after analyses of the safety outcomes of the proposed randomised clinical study.
5. This BD Odon Device is exclusively for clinical investigation only.
6. Ensure packaging is not damaged prior to use. Discard product if packaging is found to be damaged.

General Precautions

As with other devices for assisted vaginal birth the following precautions should be taken before applying the BD Odon Device to ensuring that conditions for safe application of device are met:

- Verify full dilation of cervix, fetal head station at the ischial spines or below
- Verify cephalic vertex presentation (Occiput anterior, Occiput posterior, Occiput transverse positions)
- Verify rupture of membranes
- Provide adequate analgesia according to facility procedures
- Position women in lithotomy position
- Empty bladder
- Re-confirm fetal position
- Lubricate the birth canal

Symbol Legends

STERILE R	Sterilized using radiation
	Do not re-use
	Do not re-sterilize
	Do not use if package is damaged
	Fragile, handle with care
	Keep away from sunlight
	Keep dry
LATEX	Contains or presence of natural rubber latex
LOT	Batch code
	Use-by date
	Date of manufacture
	Manufacturer
EC REP	Authorized representative in the European Community
i	Consult instructions for use
	Caution
SN	Serial number

Manufacturer: Becton Dickinson Medical Products Pte Ltd, 30 Tuas Avenue 2, Singapore 639461

EC REP Authorised representative in the European Community:
Becton Dickinson France SAS, 11 rue Aristide Bergès, ZI des Iles, 38800 Le Pont de Claix, France

Odon Device is a trademark of Air Bag One. BD, the BD Logo, and all other trademarks are property of Becton, Dickinson and Company. © 2017 BD

VENDOR INFO:

-HOLDING LINES ONLY... DO NOT PRINT.
NOT USE FOR DIMENSIONAL PURPOSES
-IFU DIMENSIONS TO BE 210MM X 297MM.

PMS2755

BD

BDMP R&D
30 TUAS AVENUE 2 S(639461)
SINGAPORE

THIS MATERIAL IS THE CONFIDENTIAL AND PROPRIETARY PROPERTY OF BECTON DICKINSON AND COMPANY. NO PORTION MAY BE USED, REPRODUCED OR DISTRIBUTED WITHOUT PRIOR WRITTEN PERMISSION.

REV (VER)	01(A)
CR NO.	CM500000111539
REVISION DATE	-
TITLE	BD Odon Device™ - Instruction For Use
DESIGNED BY	Narayanan V
DRAFTED BY	Narayanan V
CREATION DATE	24 NOV 2017
UNITS	METRIC/INCHES UNLESS OTHERWISE SPECIFIED
SCALE	Scale Size 1:1 NOT PRINTED TO SCALE
SHEET	1 OF 2
TYPE	EXPERIMENTAL
DRAWING NUMBER	SRD-DGP0036
FILE NAME	SRD-DGP0036_rev 01

Document: SRD-DGP0036 Doc Type: ZLB Status: Released EFFECTIVE
Valid From: 07-Dec-2017 To: 31-Dec-9999 Doc Part: EN Revision: 01 Change #: 500000111539
Print Date: 07-Dec-2017 05:24:29 GMT Standard Time Usage: Prototype Usage Version: A Classification: Restricted

DEVICE USE STEPS (FOR CLINICAL INVESTIGATION ONLY)

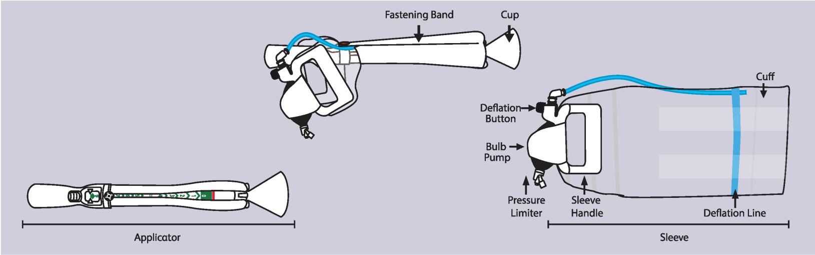

1. Ensure conditions for safe application of device are met:
   a. Full dilation of cervix, fetal head 0 to + 3 station, cephalic vertex presentation (OA, OP, OT positions), rupture of membranes
   b. Provide adequate analgesia according to facility procedures
   c. Position women in lithotomy position
   d. Empty bladder
   e. Re-confirm fetal position
   f. Lubricate the birth canal

2. Remove BD Odon Device from packaging without compromising the sterility of the device.

3. Pull back the fastening band until the blue deflation line is exposed.

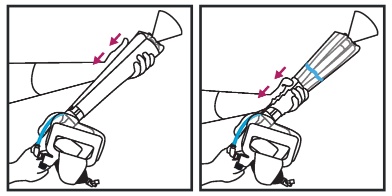

4. With the fastening band still intact, generously lubricate the inside and outside of the sleeve and the cup.

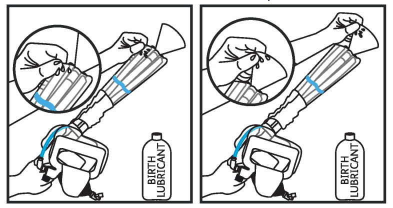

5. While holding the applicator handle gently slide the fastening band back to the top of the sleeve.

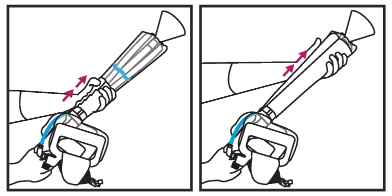

6. Grip the applicator handle and ensure the viewing window is facing upwards.

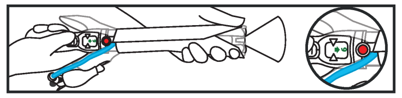

7. Fold the cup and gently insert it through the vulva and check it has regained its circular shape.

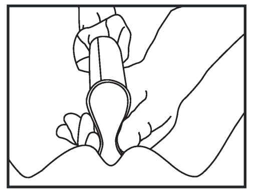

8. Check that there is no maternal tissue trapped between the cup and the fetal head.

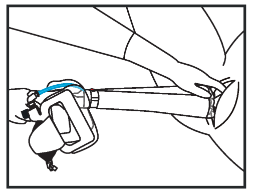

9. With your hand on the handle of the applicator, gently push the device through the vulva. Use your other hand to guide the device and do not apply any force with it.

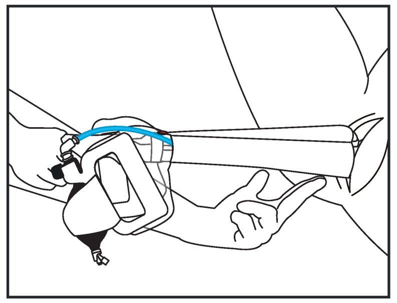

A

DEVICE USE STEPS (FOR CLINICAL INVESTIGATION ONLY)

10. Unfasten the red button

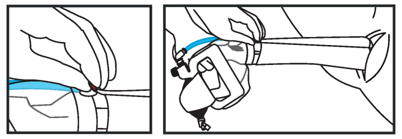

11. Open and completely remove the fastening band.

Note: ensure the sleeve and applicator remain in place inside the vulva.

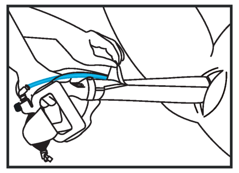

12. Between contractions, keeping both hands away from the sleeve, continue to gently push the applicator, starting at 45˚ below the horizontal and following the curvature of the birth canal. Monitor progress by looking through the viewing window.

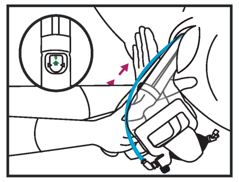

13. Continue to insert the device and stop when "0" appears in the viewing window.

Note: device is fully inserted when "0" appears in the viewing window.

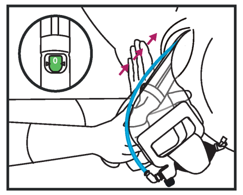

14. Squeeze the bulb pump fully and firmly at least 8 times to inflate the cuff.

Note: There is a pressure limiter in the bulb which prevents over inflation of the cuff.

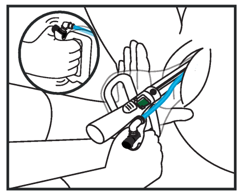

15. While protecting the perineum with one hand, use the other hand to completely withdraw the applicator and cup, leaving only the sleeve in place.

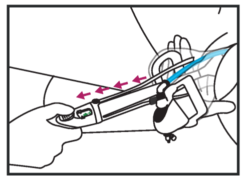

16. To compensate for possible reduction in cuff pressure, squeeze the bulb pump fully and firmly 2 more times prior to traction.

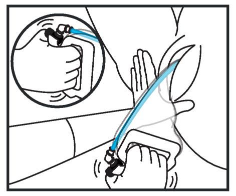

17. Grasp the sleeve handle, and during contractions pull gently and progressively following the J-shape of the birth canal.

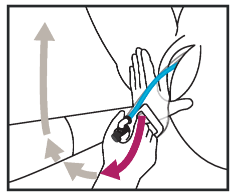

18. While continuing to pull gently along the J-shape of the birth canal, confirm the fetal head is descending with pulling efforts.

Note: if baby is not delivered at the first contraction repeat steps 16, 17 and 18 with any subsequent contractions.

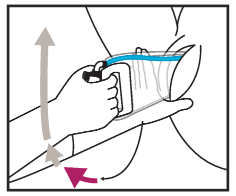

19. Once you see the blue deflation line completely (approximately at crowning) deflate the cuff by pushing on the blue deflation button. Pull the sleeve handle continuing to press the deflation button following the J-shape of the birth canal.

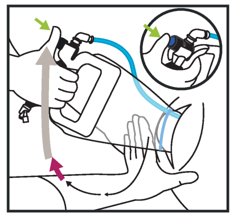

20. Continue to pull the sleeve handle while pressing the blue deflation button to pull the fetal head until the sleeve detaches from the head.

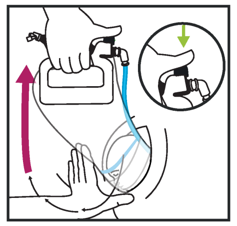

21. Proceed to assist the birth of the baby as per normal procedure.

22. Discard disposable applicator and sleeve according to local appropriate procedure. Do not reuse.

B

BD

BDMP R&D
30 TUAS AVENUE 2 S(639461)
SINGAPORE

THIS MATERIAL IS THE CONFIDENTIAL AND PROPRIETARY PROPERTY OF BECTON DICKINSON AND COMPANY. NO PORTION MAY BE USED, REPRODUCED OR DISTRIBUTED WITHOUT PRIOR WRITTEN PERMISSION.

REV (VER) 01(A)	TITLE BD Odon Device™ - Instruction For Use			
CR NO. CM500000111539	DESIGNED BY Narayanan V	UNITS METRIC/INCHES UNLESS OTHERWISE SPECIFIED	SHEET 2	TYPE EXPERIMENTAL
REVISION DATE -	DRAFTED BY Narayanan V		OF	
	CREATION DATE 24 NOV 2017	SCALE Scale Size 1:1 NOT PRINTED TO SCALE	2	DRAWING NUMBER SRD-DGP0036
	FILE NAME SRD-DGP0036_rev 01			

VENDOR INFO:

-HOLDING LINES ONLY... DO NOT PRINT.
NOT USE FOR DIMENSIONAL PURPOSES
-IFU DIMENSIONS TO BE 210MM X 297MM.

PMS2755

Document: SRD-DGP0036
Valid From: 07-Dec-2017 To: 31-Dec-9999
Print Date: 07-Dec-2017 05:24:29 GMT Standard Time

Doc Type: ZLB
Doc Part: EN
Usage: Prototype Usage

Status: Released EFFECTIVE
Revision: 01 Change #: 500000111539
Version: A Classification: Restricted
