# Supplementary material for: Novel device for assisted vaginal birth: using integrated qualitative case study methodology to optimise Odon Device use within a feasibility study in a maternity unit in the Southwest of England
Source: BMJ Open. 2022 Aug 4;12(8):e059115. doi: 10.1136/bmjopen-2021-059115 (PMC9358957; doi:10.1136/bmjopen-2021-059115)
Supplement: Author's manuscript [file bmjopen-2021-059115.draft_revisions.pdf]

BMJ Open is committed to open peer review. As part of this commitment we make the peer review history of every article we publish publicly available.

When an article is published we post the peer reviewers' comments and the authors' responses online. We also post the versions of the paper that were used during peer review. These are the versions that the peer review comments apply to.

The versions of the paper that follow are the versions that were submitted during the peer review process. They are not the versions of record or the final published versions. They should not be cited or distributed as the published version of this manuscript.

BMJ Open is an open access journal and the full, final, typeset and author-corrected version of record of the manuscript is available on our site with no access controls, subscription charges or pay-per-view fees (http://bmjopen.bmj.com).

If you have any questions on BMJ Open's open peer review process please email info.bmjopen@bmj.com

BMJ Open

Novel device for assisted vaginal birth: using integrated qualitative case study methodology to optimise Odon Device use within a feasibility study in a maternity unit in the Southwest of England

Journal:	*BMJ Open*
Manuscript ID	bmjopen-2021-059115
Article Type:	Original research
Date Submitted by the Author:	08-Nov-2021
Complete List of Authors:	Hotton, Emily; University of Bristol; North Bristol NHS Trust<br>Bale, Nichola; North Bristol NHS Trust<br>Draycott, Tim ; North Bristol NHS Trust<br>Blencowe, Natalie; University of Bristol, Centre for Surgical Research, School of Social and Community Medicine<br>Lenguerrand, Erik; University of Bristol School of Clinical Science, School of Clinical Sciences, Musculoskeletal Research Unit<br>Crofts, Joanna; North Bristol NHS Trust<br>Wade, Julia; University of Bristol, Bristol Medical School
Keywords:	QUALITATIVE RESEARCH, OBSTETRICS, STATISTICS & RESEARCH METHODS
Note: The following files were submitted by the author for peer review, but cannot be converted to PDF. You must view these files (e.g. movies) online.	
S2 Video.MOV	

SCHOLARONE™
Manuscripts

I, the Submitting Author has the right to grant and does grant on behalf of all authors of the Work (as defined in the below author licence), an exclusive licence and/or a non-exclusive licence for contributions from authors who are: i) UK Crown employees; ii) where BMJ has agreed a CC-BY licence shall apply, and/or iii) in accordance with the terms applicable for US Federal Government officers or employees acting as part of their official duties; on a worldwide, perpetual, irrevocable, royalty-free basis to BMJ Publishing Group Ltd ("BMJ") its licensees and where the relevant Journal is co-owned by BMJ to the co-owners of the Journal, to publish the Work in this journal and any other BMJ products and to exploit all rights, as set out in our licence.

The Submitting Author accepts and understands that any supply made under these terms is made by BMJ to the Submitting Author unless you are acting as an employee on behalf of your employer or a postgraduate student of an affiliated institution which is paying any applicable article publishing charge ("APC") for Open Access articles. Where the Submitting Author wishes to make the Work available on an Open Access basis (and intends to pay the relevant APC), the terms of reuse of such Open Access shall be governed by a Creative Commons licence – details of these licences and which Creative Commons licence will apply to this Work are set out in our licence referred to above.

Other than as permitted in any relevant BMJ Author's Self Archiving Policies, I confirm this Work has not been accepted for publication elsewhere, is not being considered for publication elsewhere and does not duplicate material already published. I confirm all authors consent to publication of this Work and authorise the granting of this licence.

For peer review only

Novel device for assisted vaginal birth: using integrated qualitative case study methodology to optimise Odon Device use within a feasibility study in a maternity unit in the Southwest of England

Emily J Hotton[1,2 *], Natalie S Blencowe[3,4], Nichola Bale[2], Erik Lenguerrand[1,2], Tim J Draycott[2], Joanna F Crofts[2], Julia Wade[5]

[1] Translational Health Sciences, University of Bristol, Bristol, UK

[2] Southmead Hospital, North Bristol NHS Trust, Bristol, UK

[3] University Hospitals Bristol NHS Foundation Trust, Bristol, UK

[4] Centre for Surgical Research, Population Health Sciences, University of Bristol, Bristol, UK

[5] Population Health Sciences, University of Bristol, Bristol, UK

*** Corresponding Author:**

Women and Children's Academic Research

The Chiltern's

Maternity Unit

Southmead Hospital

Bristol

United Kingdom

BS10 5NB

Tel: 0117 414 6764

Email: emily.hotton@nhs.net

Running title: Optimising the use of the Odon Device

Abstract word count: 300

Word count: 3939

For peer review only - http://bmjopen.bmj.com/site/about/guidelines.xhtml

Objective

When novel devices are used ‘in human’ for the first time, their optimal use is uncertain because clinicians only have experience from pre-clinical studies. This study aimed to investigate factors that might optimise use of the Odon Device for assisted vaginal birth.

Design

We undertook qualitative case studies within the ASSIST Study, a feasibility study of the Odon Device. Each ‘case’ was defined as one use of the device and included at least one of the following: observation of the attempted assisted birth, and an interview with the obstetrician, midwife or woman. Data collection and thematic analysis ran iteratively and in parallel.

Setting

Tertiary referral NHS maternity unit in the Southwest of England.

Participants

Women requiring a clinically indicated assisted vaginal birth.

Intervention

The Odon Device, an innovative device for assisted vaginal birth.

Primary and secondary outcomes measures

Determining the optimal device technique, device design and defining clinical parameters for use.

Results

Thirty-nine cases involving an attempted Odon assisted birth were included in this study, of which 19 resulted in a successful birth with the device. Factors that improved use included optimisation of device technique, device design and clinical parameters for use. Technique adaptations included: applying the device during, rather than between, contractions; having a flexible approach to the application angle; and deflating the air cuff sooner than originally

For peer review only

proposed. Three design modifications were proposed involving the deflation button and sleeve. Although use of the device was found to be appropriate in all fetal positions, it was considered contraindicated when the fetal station was at the ischial spines.

Conclusions

Case study methodology facilitated the acquisition of rapid insights into device function in clinical practice, providing key insights regarding use, design, and key clinical parameters for success. This methodology should be considered whenever innovative devices are introduced into clinical practice.

Trial registration

ASSIST Study registration: ISRCTN10203171 https://doi.org/10.1186/ISRCTN10203171

Keywords

Assisted vaginal delivery, qualitative research, translational research, Medical Devices

For peer review only

Article Summary

- Case study methodology was successfully used in an intrapartum setting to evaluate the use of a novel device, the Odon Device, for assisted vaginal birth
- This approach enabled deep understanding of device technique, design and clinical parameters for use, and how these may have influenced the outcome of successful birth
- Iterative data analysis and feedback of findings enabled rapid dissemination of findings to key stakeholders, and consensus regarding future alterations to device design, technique, and selection criteria for optimal device use

For peer review only

For peer review only - http://bmjopen.bmj.com/site/about/guidelines.xhtml

Introduction

Each year approximately 82,000 women in the UK have an assisted vaginal birth (AVB).[1] In recent years, despite the known advantages of AVB, this rate has reduced with a corresponding increase in Caesarean births in the second stage of labour. Current devices for AVB require a high level of training and skill (with additional expertise required to define the fetal position) and can be associated with significant maternal and neonatal morbidity if used incorrectly.[2] An innovative device that is easier and safer to use, could increase women's access to AVB, which in turn would help to reduce the number of emergency Caesarean sections performed in the second stage.[3,4]

Before introducing devices into widespread practice, it is necessary to evaluate their safety and efficacy, and obtain CE marking. However, other factors – such as device technique, design, and clinical parameters for use – are not routinely assessed, yet may ultimately limit their success. Preliminary feasibility work exploring these other factors may be valuable prior to evaluation within a definitive randomised controlled trial. The Odon Device (**Figure 3**) has undergone rigorous pre-clinical[5], simulation[6,7], human factors[8] and Phase 1 first-in human investigation[9] which conclude that it appeared to be safe. However, the Odon Device has hitherto not been used in the intended population: women requiring an AVB.

This study applied qualitative case study methodology to examine in detail how the Odon Device (version 4.1) is used for AVB, and to determine what factors may impact on optimal use. The study was embedded in the ASSIST Study – a feasibility study of the Odon Device.[10,11]

Figure 1 Diagram of the Odon Device

Methods

Research design

The ASSIST Study[10,11] was conducted in a maternity unit in the Southwest of England with full detail published elsewhere.[11,12] Integrated within the study was qualitative research, using case study methodology to explore the factors that may influence optimum device

For peer review only - http://bmjopen.bmj.com/site/about/guidelines.xhtml

use. Case study methodology is particularly suited to answering ‘how and why’ questions and providing in-depth contextual detail, essential in early evaluations[13] of complex interventions,[14–16] such as use of a novel device for AVB,[17] and has previously been used to explore surgical innovation.[18] In this study, each ‘case’ was defined as one use of the device and included at least one of the following: observation of the attempted Odon assisted birth and/or an interview with the obstetrician, midwife or woman. The researcher ensured that the use of the device in the study was compared against the Instructions For Use (IFU) document, which is mandated by regulatory bodies as one of the processes to ensure device safety and efficacy. Given the focus of this paper is of the technical aspects of device use, data presented reports observation and healthcare professional interview findings. Data reporting experiences of women are presented separately.[12]

Participants

There were two groups of participants for the case studies: women and healthcare professionals (obstetricians and midwives). All women participating in the ASSIST Study were eligible to be included in the case study research and gave written consent.[12] All trained operators and midwives provided written consent. There were five operators, three consultants and two registrars.

Sampling

Typical sampling for observation was in part purposive and in part opportunistic (i.e. dependent on the researcher being on site and available to conduct the observation). The aim was to include a range of clinical indications for AVB and a range of operators.

Patient and public involvement

Patients and the public were involved in all aspects of the ASSIST Study,[10,12]

For peer review only

Data collection

Data collection for eight of the cases included observation of the attempted Odon assisted birth. Observations, including technical details, contextual factors and communication, were prospectively recorded on a bespoke observation schedule. Detailed observations of the operative steps performed by the obstetrician during AVBs were recorded enabling a stepwise account of the 'usual steps' to be generated and compared against the IFU. The original IFU were developed prior to the ASSIST study during phase 1 clinical and simulation studies and included 22 operative steps.[8,9] In these IFU, the AVB was divided into six domains according to purpose (**Table 1**). The IFU and instructional video for operators used for the Odon Device in the ASSIST Study can be viewed in Supplementary files 1 and 2.

All women who had the birth of their baby formally observed were invited to participate in an interview at day one postnatal and clinicians within five days following the assisted birth.[10] In line with usual practice in conducting case study research a flexible approach was taken to which data were collected for each case, based on the value of insights gained for each data source. Any method of data collection (observation or interview) could be suspended if it was observed to be delivering no new insights.

Data analysis

Data collection and analysis were iterative and ran in parallel using the six-step framework described for thematic analysis.[19] Data analysis was largely inductive although some deduction derived from using the IFU as a framework against which to evaluate what took place. All data for each case were read together to identify and organise codes. Codes were developed using text that captured significant views in the data, then grouped to reflect developing themes, with code descriptions and sample quotes assigned. Double coding of a proportion of interview transcripts (20%) was undertaken by JW. A narrative report was created for each case, triangulating all available data. Any issues requiring clarification were highlighted during the creation of the report, for exploration during subsequent interviews.

For peer review only - http://bmjopen.bmj.com/site/about/guidelines.xhtml

Commonality and variances across cases were discussed between the researchers and used to further shape evolving themes and sampling. This systematic analysis supported rapid within- and cross-case comparison. NVivo 12 (QSR International, Melbourne, Australia) was used to organise data and support analysis.

Feedback of findings

Iterative data collection and analysis enabled the rapid identification of key learning points or corrections to technique for dissemination to operators (see Table 2). Key findings were relayed rapidly to operators using messages via an end-to-end encryption platform, regular face-to-face discussions and operator debriefs. Furthermore, following the 36th Odon assisted birth, an interactive summit was held with key stakeholders (the clinical research team, design engineers, statisticians, and funders), with the aim of sharing learning experiences and gaining consensus regarding any changes that may be suggested.

Funding

This work was supported by the Bill & Melinda Gates Foundation [INV-010180]. Under the grant conditions of the Foundation, a Creative Commons Attribution 4.0 Generic License has already been assigned to the Author Accepted Manuscript version that might arise from this submission.

Results

Forty births were assisted with the Odon Device at North Bristol NHS Trust, UK, between October 2018 and January 2019. One case had no qualitative data because the researcher was unavailable, resulting in 39 case studies arising from 40 (97.5%) single uses of the Odon Device (**Table 2**). Nineteen births were successfully assisted with the Odon Device. Of those that were unsuccessful, 19 were assisted by forceps and two by Caesarean section. Observations varied in length from 33 to 68 minutes. Interviews with women lasted 6.5 to

For peer review only

For peer review only - http://bmjopen.bmj.com/site/about/guidelines.xhtml

9.6 minutes, interviews with operators lasted between 5.4 and 26.1 minutes and interviews with midwives lasted 3.4 to 13.2 minutes.

It became apparent that there were three factors contributing to optimisation of device use: (i) device technique, (ii) device design and (iii) clinical parameters for device use (Figure 2).

Figure 2 How case study methodology may be able to determine optimal device use through bridging multiple factors relating to the device

Device technique

Suggested adaptions to the original IFU included (i) device application during rather than between contractions, (ii) altering the application angle and (iii) deflating the air cuff as soon as any aspect of the blue deflation line became visible.

Device application with a contraction

The original IFU stated that the Odon Device should be applied between contractions, as was standard practice with forceps and ventouse. It became apparent during the first two attempted AVBs that this disimpacted the fetal head out of the pelvis and operators were unable to correctly place the device:

> D1: *'Again, I had to use significant pressure to try and get the device over the fetal head. And loads of liquor came down during the application suggesting that there was some degree of disimpaction.'*

By the third attempted birth, operators had adapted their technique to include fundal pressure to aid application, which resulted in successful device application and the first successful AVB. The use of fundal pressure, although successful, was not well tolerated by women without a regional anaesthetic:

> M3: *'Significant fundal pressure that was used at the time…she was uncomfortable…maybe that will be something up for review.'*

Following feedback from qualitative findings, the application technique was adapted again during the eighth birth. This was the first time the Odon Device was applied during a

For peer review only - http://bmjopen.bmj.com/site/about/guidelines.xhtml

For peer review only

contraction without the use of fundal pressure, resulting in a successful application and birth. Fundal pressure was only used in a small number of births and quickly dropped from the technique as soon as application with a contraction was found to be successful:

> D2: *'I haven't used fundal pressure since delivery number two or three for me, but what has been very successful is putting it on during a contraction. I think.'*

Device application angle

The original IFU stated that the device should be applied 'starting at 45° below the horizontal'. By the eighth attempted birth it was apparent that this was not optimal and operators naturally moved to a more 'horizontal' application:

> D2: *'I definitely pushed the device in at a much flatter angle, much more parallel with the bed than I had in the past...'*

All operators quickly agreed that the angle required might be dependent on factors such as fetal position and station:

> D1: *'So I was kind of like, "Oh, OP, it might be more, you know, it could be difficult because it's an OP…"'*

> D2: *'I think we've still got to continue experimenting or changing the angle of insertion. I think there may be an optimum angle of insertion or it may be that we have to change angle of insertion for different stations...'*

Deflating the device

The original IFU stated that 'once you see the blue deflation line completely' the air cuff should be deflated. By the third attempted birth it became apparent to the observer that this was too late and that the optimum time for air cuff deflation seemed to be when any section of the blue line could be seen:

> O5: *'Noticed that it was not the anterior blue deflation line that the operator was looking at the deflate but the posterior deflation line, due to the fact that there is an acute J curve and anterior line not seen. Will need to change this in training.'*

For peer review only

These observations were fed back rapidly and iteratively to the Odon Device operators and further discussed with the wider research team at the Odon Summit (Table 2).

Device design and performance

Multiple potential device adaptations were noted during the case study research. Four design modifications for future device adaptations were identified: (i) strengthening the sleeve seal lines, (ii) creating a wider opening between the sleeve handles, (iii) altering the design of the deflation button and (iv) address the manufacturing fault that was identified.

Sleeve seal lines and opening between sleeve handles

One operator noted that the sleeve seal lines tore during traction, on several occasions:

> D4: *'… the sleeve is not sturdy… it might actually rip it open, which has happened with me a few times.'*

During device inspection, it was noted that all devices had small tears (<2cm) in the seal lines of the sleeve, and one had a significant tear (>7cm). There was no evidence any of these tears had had a negative effect on the function of the Odon Device, indeed the device with a significant tear achieved a successful Odon birth. Tearing was thought to have occurred when operators opened the sleeve handles before and between tractions to physically look at the station of the vertex. In contrast to standard devices used for AVB, there was little proprioceptive feedback to ascertain the station of the baby, so visual inspection was useful:

> O5: *'…I got the impression that the operator was unsure as to whether the head had descended so opened the handles to look inside the sleeve.'*

Following interviews, it was suggested that the opening between the two handles was made wider to enable operators to view the progression of the baby's head more easily (Figure

For peer review only

For peer review only - http://bmjopen.bmj.com/site/about/guidelines.xhtml

S1). Ultrasound assessment was not used as this method was not routinely adopted in our unit at the time of the study.

Deflation button

In six cases it was noticed that the operator accidentally pressed the deflation button. Each time this occurred, the cuff was reinflated immediately. All operators agreed that the design of the deflation button should be altered to reduce the risk of inadvertent activation (Figure S2):

> O3: '*Operator accidentally pressed the deflation button 'oh, whoops that was my fault, I'll just re-inflate'.'*

Manufacturing fault

All devices were disinfected and inspected following their use as per protocol.[10,11] During this inspection, four devices were found to have an ineffective bulb pump (**Error! Reference source not found.**) which resulted in inadequate cuff inflation (**Table 2**). Operators' comments during the attempted births reflected this, as the device did not act in the expected manner.

> D4: *'Yes, there was no grip… It just came out deflated, so it didn't feel right.'*

This prompted a rapid retrospective review of all used and stored devices to ensure that no other unsuccessful attempts were attributed to this fault, none were.

Optimal clinical parameters for Odon Device use

The Odon Device was used to successfully assist births in all fetal positions. Midwives particularly noted how the device could help deliver a baby in the occipito-posterior position which is a technically more challenging position:

For peer review only

> M9: *'I think, probably, it could be quite universal as an instrumental device. It didn't seem to matter whether the baby was OA* [occipito-anterior] *or* OP [occipito-posterior]...'

However, although the device could be successful at assisting birth in all positions, it became apparent that for women with fetal station at spines or a more complex presentation (such as brow or nuchal arm) the device was not successful. Operators were either unsuccessful at applying the device correctly onto the fetal head or the device simply slipped off the fetal head with the initial traction:

> *D1: 'So, it was direct OP at the spines, and it was almost coming to a brow, I could feel the orbital ridges…I was thinking, "Oh, I'm really not sure that this is going to work."…I didn't feel that was a failed Odon, that was a baby that was never going to come out vaginally.'* [unsuccessful Odon-failed rotational forceps, emergency Caesarean section]

As experience with the device increased, it became apparent that the device could be used comfortably without a regional anaesthetic (with only perineal infiltration of local anaesthetic). Device use was noted to be better tolerated than bladder emptying by urethral catheterisation, a procedure that is less invasive:

> D2: *'She actually found the catheterisation more uncomfortable than putting on the Odon Device with no analgesia at all.'*

Feedback to operators

All qualitative case study findings relating to device technique, design, and clinical parameters for use were presented to key stakeholders at the Odon Summit by the qualitative researcher. The case study research provided suggestions for device technique adaptation for some of the operative steps, but not for them all. It was agreed that there were still unanswered questions regarding the technique (such as which angle to use for application) and that further data was required to achieve this. Clinically important adaptations to device design were agreed upon (including altering the deflation button

For peer review only

For peer review only - http://bmjopen.bmj.com/site/about/guidelines.xhtml

design) and the clinical parameters for use were confirmed, with an agreement that the device should not be used if the vertex is at the level of the ischial spines.

Discussion

Case study research identified three areas that could optimise device use: (i) device technique, (ii) device design and (iii) acceptable clinical parameters. Principal technique adaptations were centred on device application and deflation of the air cuff. The initial IFU specified a particular angle for device application however, during clinical use it became apparent that this angle needed to be flexible and was less acute than originally specified however, there was no consensus on the exact optimal angle and it was surmised that more data would be required to achieve this. Device modifications of altering the sleeve and deflation button were recommended for useability rather than to transform the functionality of the device. The manufacturing fault was quickly identified and rectified by the manufacturer through post-use device inspection. Optimal parameters for device use were proposed and focussed primarily on the station of the baby, with use at station spines recommended to be prohibited. Adaptations to optimise device use were adopted by the manufacturer to create Odon Device (version 4.2) which was used in two further Odon Device feasibility studies, each studying 104 Odon assisted births. These have recently closed to recruitment in the UK[20] and France[21] and aimed to address the unanswered aspects of optimal device use, specifically the technique. These findings will be published once follow-up and data analysis in complete. Case study research enabled systematic, rapid generation of data and understanding of device use that enabled the researchers and manufacturers develop study protocols and device updates to support the ongoing investigation of the device.

Strengths and limitations

This was the first time that research has been undertaken on the Odon Device in clinically indicated cases and indeed the first-time case study research has been used to explore the use of devices for AVB. Device design and technique is unique to the device and although cannot be directly compared to other devices for AVB step-by-step, some comparisons and differences can be noted. The Odon Device, unlike other devices for AVB[22], can only be successfully applied during a contraction or with maternal effort, even though techniques

For peer review only - http://bmjopen.bmj.com/site/about/guidelines.xhtml

358 for traction once the device is applied appear similar. Clinical indications for use are slightly different to that of forceps and ventouse in the UK.[22] In the UK, all currently used devices for AVB are permitted to be used at stations spines or below. We have demonstrated that this is not the case for the Odon Device, as we have demonstrated that this will not be successful. Interestingly, performing AVBs at station spines is not permitted in other countries.[23]

An AVB is a complex intervention, and this makes studying the use of the device challenging. Qualitative case study methodology has been used to explore technique in surgical procedures[18] however, there are no published examples of case study methodology being used to investigate novel devices. The case studies integrated participant observation as well as interviews with operators, midwives, and women to explore the introduction of an innovative device in context and in detail. The benefits of this were that experiences, and views of all stakeholders were easily obtained, and we were able to investigate operator views in detail. Triangulation of data linked to a particular case led to insights for amendments for optimum device use being identified more rapidly that if a single source of qualitative data (e.g., observation or interview only) had been used. Rapid dissemination of findings resulted in prompt adoption of beneficial techniques for use. By using this methodology and incorporating data from all stakeholders (operators, midwives, and women) and observations we were able to gain a balanced and comprehensive assessment of the use of the device. When trying to understand optimal device use, operator interviews were found to be of crucial importance. Comparing case study data collected under different conditions (such as different analgesia, different presentations of babies, different operators) enabled commonalities and disparities in technique to be highlighted and thoroughly investigated. This enabled the clinical research team to propose evidence-based modifications to the device design and provide clarity on recommendations for clinical parameters for use. Case study methodology encouraged operators to reflect, critique and appraise their use of the device for each birth, resulting in enhanced and enriched communication between operators regarding their experiences through conversations and a dedicated operator messaging group. In future, data from encrypted social media platforms could be incorporated into the qualitative data for analysis.

For peer review only

Reporting was undertaken following the Standards for Reporting Qualitative Research[34] (Supplementary information 3).

There are limitations to this study. The aim of understanding the optimal operative steps for device use, and thus confirming a finalised IFU were not met. For some operative steps consensus was reached as to the recommended course of action (such as applying the device with a contraction). However, for others more data were required (such as what specific angle of application to use). Case studies within the ASSIST Study were finite. Observations were undertaken where possible, however due to the unpredictable nature of AVBs it was not possible to attend all assisted births. Indeed, none of the more complex attempted AVBs performed in the operating theatre were observed. This could have an impact on the generalisability of the findings as births undertake in the operating theatre are often more technically challenging for operators. All interviews with clinicians were undertaken within five days following the assisted birth. Recollections of the clinicians may have been less accurate the longer the time between assisted birth and interview. The case studies were undertaken by a specialist trainee in obstetrics and gynaecology meaning that pre-conceptions and existing knowledge may have influenced the collection and interpretation of the data, although at the time of commencing the case studies the researcher was naïve to the use of the Odon Device in the clinical setting. Lastly, operators may have changed their behaviours during observations, perhaps not reflecting their real-life practice.

There are two key next steps that should be considered. Firstly, feasibility of the use of the Odon Device for AVB should be undertaken in different healthcare settings. Thus far, research has been undertaken in high-income settings where AVB is used regularly. Exploring device using in low- and middle- income settings, including where rates of AVB are lower than the UK and France could help understand if there are further considerations for optimal device use that need to be addressed. Secondly, following the completion of the two further feasibility studies, a decision needs to be made as to whether the device is ready to be compared against available alternatives (forceps and ventouse) in a randomised controlled trial. As recommended by IDEAL-D,[24] researchers need to be satisfied that the

For peer review only - http://bmjopen.bmj.com/site/about/guidelines.xhtml

technique, design and clinical parameters for use are sufficiently stable to enable this to happen.

Conclusion

Case study methodology facilitated insights into optimal technique, design and clinical parameters for use of the Odon Device. Optimising use of a device is an essential prerequisite to evaluating outcomes, as it will impact directly on those outcomes and may result in lower-than-expected success rates. There were two clear factors that enhanced operator communication. Firstly, systematic triangulation of data from varying data sources provided a comprehensive, contextual overview of device use and rapid understanding of amendments required and secondly, rapid feedback of insights as they emerged to operators. This also facilitated operator consensus building, which was key in understanding and developing the iterative adaptations to the device technique, design and clinical parameters for device use. This is of paramount importance for getting operator buy-in for the next steps of device evaluation. This methodology should be considered whenever innovative devices are introduced to clinical trials and settings. It allows for rapid assessment of device use and can support timely iterative adaptions to ensure there are minimal delays between device use in research and adoption in clinical practice.

For peer review only

Acknowledgements

The authors would like to thank all the women who agreed to take part in this research and all the maternity staff who enabled to safe completion of the ASSIST Study. The research was affiliated with but not funded by the NIHR Biomedical Research Centre at University Hospitals Bristol and Weston NHS Foundation Trust and the University of Bristol. The views expressed are those of the author(s) and not necessarily those of the NIHR or the Department of Health and Social Care, and the MRC ConDuCT-II (Collaboration and innovation for Difficult and Complex randomised controlled Trials In Invasive procedures) Hub for Trials Methodology Research (MR/K025643/1).

For peer review only - http://bmjopen.bmj.com/site/about/guidelines.xhtml

Competing interests

Authors report no competing interests.

Contribution to Authorship

EJH, NSB and JW developed the concept for the study. EJH performed all data collection and analysis with co-coding performed by JW. EJH wrote the initial draft of the manuscript, with support from NSB, JFC and JW. EJH, NSB, NB, EL, TJD, JFC and JW reviewed and approved the final manuscript.

Ethical approval

The research was approved by South Central–Berkshire REC, UK on 3rd September 2018 (18/SC/0344), the MHRA on 9th August 2018 and the HRA on 3rd September 2018.

Data sharing statement: Data are available upon reasonable request.

For peer review only

Table/figure caption list

Table 1 Original components of application of the Odon Device for an assisted vaginal birth

Table 2 Summary of 40 cases investigating the Odon Device with adaptations made to device technique

Figure 3 Diagram of the Odon Device

Figure 4 How case study methodology may be able to determine optimal device use through bridging multiple factors relating to the device

Supplementary file (S1) Odon Device IFU for Clinical Studies

Supplementary file (S2) Instructional video for the ASSIST Study

Supplementary file (S3) Standards for Reporting Qualitative Research (SRQR)

For peer review only

References

1. Digital N. NHS Maternity Statistics - England, 2013-14 [Internet]. 2015 [cited 2021 Oct 11]. Available from: http://digital.nhs.uk/catalogue/PUB16725

2. Hotton E, O'Brien S, Draycott TJ. Skills training for operative vaginal birth. Best Pract Res Clin Obstet Gynaecol. 2018;56:11–22.

3. Requejo JH, Belizán JM. Odon device: a promising tool to facilitate vaginal delivery and increase access to emergency care. Reprod Health. 2013;10(1):42.

4. Draycott TJ, Renzo GCD. The role of operative vaginal birth in the 21st century and a way forward. BJOG. 2017;124 Suppl 4:5–6.

5. Lange C de, Saugstad OD, Solberg R. Assessment of cerebral perfusion with contrast-enhanced ultrasound during constriction of the neck mimicking malposition of the BD Odon Device™: a study in newborn piglets. BJOG: An International Journal of Obstetrics & Gynaecology. 2017 Sep;124 Suppl 4(4 Pt 1):26–34.

6. O'Brien SM, Winter C, Burden CA, Boulvain M, Draycott TJ, Crofts JF. Fetal head position and perineal distension associated with the use of the BD Odon Device™ in operative vaginal birth: a simulation study. BJOG: An International Journal of Obstetrics & Gynaecology. 2017 Sep;124(11):10–8.

7. O'Brien SM, Winter C, Burden CA, Boulvain M, Draycott TJ, Crofts JF. Pressure and traction on a model fetal head and neck associated with the use of forceps, Kiwi™ ventouse and the BD Odon Device™ in operative vaginal birth: a simulation study. BJOG. 2017;124(11):19–25.

8. O'Brien SM, Mouser A, Odon JE, Winter C, Draycott TJ, Sumitro T, et al. Design and development of the BD Odon DeviceTM : a human factors evaluation process. BJOG: An International Journal of Obstetrics & Gynaecology. 2017;124 Suppl 4:35–43.

9. Schvartzman JA, Krupitzki H, Merialdi M, Betrán AP, Requejo J, Nguyen MH, et al. Odon device for instrumental vaginal deliveries: results of a medical device pilot clinical study. Reprod Health. 2018;15(1):45.

10. Hotton EJ, Lenguerrand E, Alvarez M, O'Brien S, Draycott TJ, Crofts JF, et al. Outcomes of the novel Odon Device in indicated operative vaginal birth. Am J Obstet Gynecol. 2021;224(6):e607.

11. O'Brien S, Hotton EJ, Lenguerrand E, Winter C, Draycott TJ, Crofts JF, et al. The ASSIST Study - The BD Odon Device for assisted vaginal birth: a safety and feasibility study. Trials. 2019;20(1).

For peer review only - http://bmjopen.bmj.com/site/about/guidelines.xhtml

12. Hotton EJ, blencowe NS, Lenguerrand E, Draycott TJ, Crofts JF, Wade J. The Odon Device to assist vaginal birth: Observations of and insights into women's experiences as narrated by health professionals and women. BMJ Open. 2021;(Submitted, under review).

13. Yin RK. Case Study Research. 5th ed. SAGE Publications Ltd; 2014. (SAGE Publications Ltd).

14. Craig P, Dieppe P, Macintyre S, Michie S, Nazareth I, Petticrew M. Developing and evaluating complex interventions: The new Medical Research Council guidance. Int J Nurs Stud. 2013;50(5):587–92.

15. Campbell M, Fitzpatrick R, Haines A, Kinmonth AL, Sandercock P, Spiegelhalter D, et al. Framework for design and evaluation of complex interventions to improve health. BMJ. 2000;321(7262):694.

16. Craig P, Dieppe P, Macintyre S, Michie S, Nazareth I, Petticrew M, et al. Developing and evaluating complex interventions: the new Medical Research Council guidance. BMJ. 2008;337:a1655.

17. Paparini S, Green J, Papoutsi C, Murdoch J, Petticrew M, Greenhalgh T, et al. Case study research for better evaluations of complex interventions: rationale and challenges. Bmc Med. 2020;18(1):301.

18. Blencowe NS, Blazeby JM, Donovan JL, Mills N. Novel ways to explore surgical interventions in randomised controlled trials: applying case study methodology in the operating theatre. Trials. 2015;16:589.

19. Braun V, Clarke V. Using thematic analysis in psychology. Qualitative Research in Psychology. 2006;3(2):77–101.

20. Hotton EJ, Alvarez M, Lenguerrand E, Wade J, Blencowe NS, Draycott TJ, et al. The Odon Device™ for assisted vaginal birth: a feasibility study to investigate safety and efficacy-The ASSIST II study. Pilot Feasibility Stud. 2021;7(1):72.

21. Mottet N, Bourtembourg A, Eckman-Lacroix A, Forner O, Mougey C, Metz JP, et al. [Focus on the Odon Device™: Technical improvements, mechanical principles and progress of the clinical research program]. Gynecol Obstetrique Fertilite Senologie. 2020;48(11):814–9.

22. Murphy D, Strachan B, Bahl R, RCOG. Assisted Vaginal Birth. BJOG. 2020;127(9):e70–112.

23. Bulletins—Obstetrics C on P. ACOG Practice Bulletin No. 154 Summary: Operative Vaginal Delivery. Obstetrics & Gynecology. 2015 Nov;126(5):1118–9.

24. Sedrakyan A, Campbell B, Merino JG, Kuntz R, Hirst A, McCulloch P. IDEAL-D: a rational framework for evaluating and regulating the use of medical devices. Bmj. 2016 Jun 9;353:i2372.

For peer review only

For peer review only - http://bmjopen.bmj.com/site/about/guidelines.xhtml

For peer review only

For peer review only - http://bmjopen.bmj.com/site/about/guidelines.xhtml

Table 1 Original components of application of the Odon Device for an assisted vaginal birth

Component	Steps within component
Preparation	Checking clinical pre-requisites for AVB<br>Lubricating the device
Device application	Removing the fastening band<br>Applying the device onto a fetal head
Cuff inflation	Ensuring the cuff is fully inflated in the correct position on the fetal head
Applicator removal	Removing the applicator from the fetal head
Traction	Following the J-shape curve of the pelvis applying traction with contractions
Removal of device	Deflating the air cuff as the fetal head is crowning

For peer review only

Table 2 Summary of 40 cases investigating the Odon Device with adaptations made to device technique

Case study no.	Successful (S) Unsuccessful (U) AVB with Odon and mode of birth	Observation	Interviews			Device issues
			Women	Operator	Midwife	
1	U – Forceps	O1	W1	D1	M1	
2	U – Forceps			D2	M2	
Fundal pressure during device application tried						
3	S – Odon			D2	M2<br>M3	
Deflation of the air cuff when only part of the blue line was seen introduced						
4	S – Odon			D2	M4	
5	U – Forceps			D1	M2	
6	S – Odon			D2*		
7	U – Forceps	O2	W2	D1	M5	AD
Accidental pressing of the deflation button first noted						
Altered the angle of device insertion						
Application during a maternal contraction introduced, use of fundal pressure removed						
8	S – Odon	O3	W3	D2	M6	AD
Opened the sleeve handles during descent to monitor progression of fetal head first noted						
9	U – Forceps	O4	W4	D1		
10	S – Odon			D2	M7	
11	S – Odon	O5	W5	D1	M8	AD
12	S – Odon	O6	W6	D1	M9	AD
13	S – Odon	O7	W7	D3	M2	
14	U – Failed rotational forceps, emergency Caesarean section			D1	M7	AD
15	U – Forceps			D2	M4	
16	U – Forceps	O8	W8	D2	M10	IBP
17	S – Odon			D1		
18	S – Odon			D3	M11	
19	S – Odon			D4		SST
20	U – Rotational forceps			D4		IBP
21	U – emergency Caesarean section			D3	M6	
22	U – Forceps			D4		
23	U – Forceps			D1	M6	IBP
24	S – Odon			D1		
25	U – Forceps			D4		
26	U – Forceps			D4		
27	S – Odon			D1		AD
28	U – Forceps			D1		
29	S – Odon			D5		
30	U – Forceps			D1		
31	U – Forceps			D4		IBP
32	U – Forceps			D1		
33	S – Odon			D5		
34	S – Odon			D2		
35	S – Odon			D2		
36	U – Forceps			D4		
Odon summit held						
37	U – Forceps			D1		
38	S – Odon			D4		
39	S – Odon			D4	M6	
40	U – Forceps			D3		

*Qualitative interview from Obstetrician not obtained for this birth
O = observation, W = woman, D = Obstetrician, M = Midwife
AD = accidental deflation, IBP = ineffective bulb pump, SST = significant sleeve tear
Bold italic steps = key stages in the study that impacted on technique

For peer review only - http://bmjopen.bmj.com/site/about/guidelines.xhtml

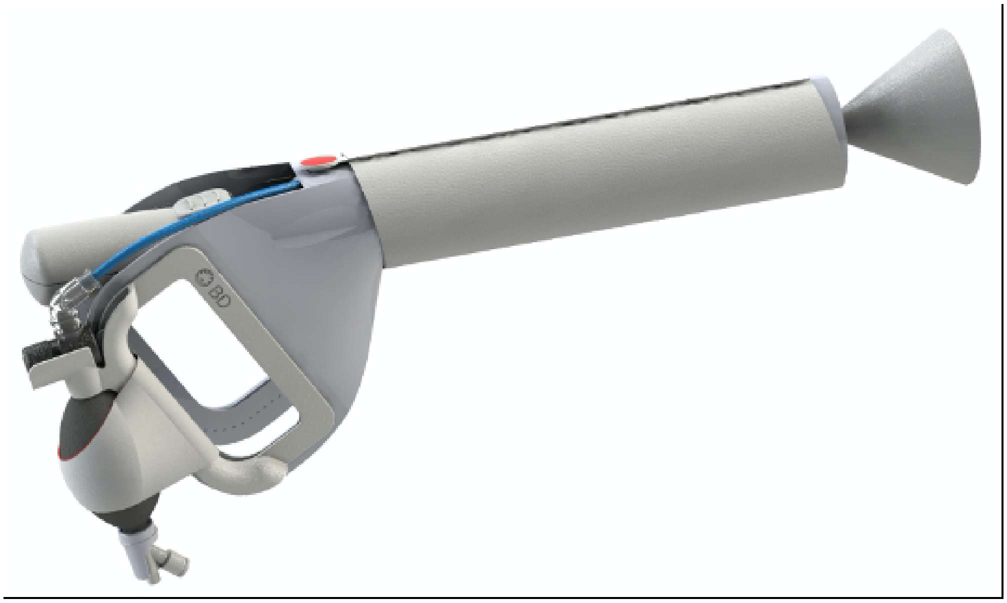

Diagram of the Odon Device

94x56mm (144 x 144 DPI)

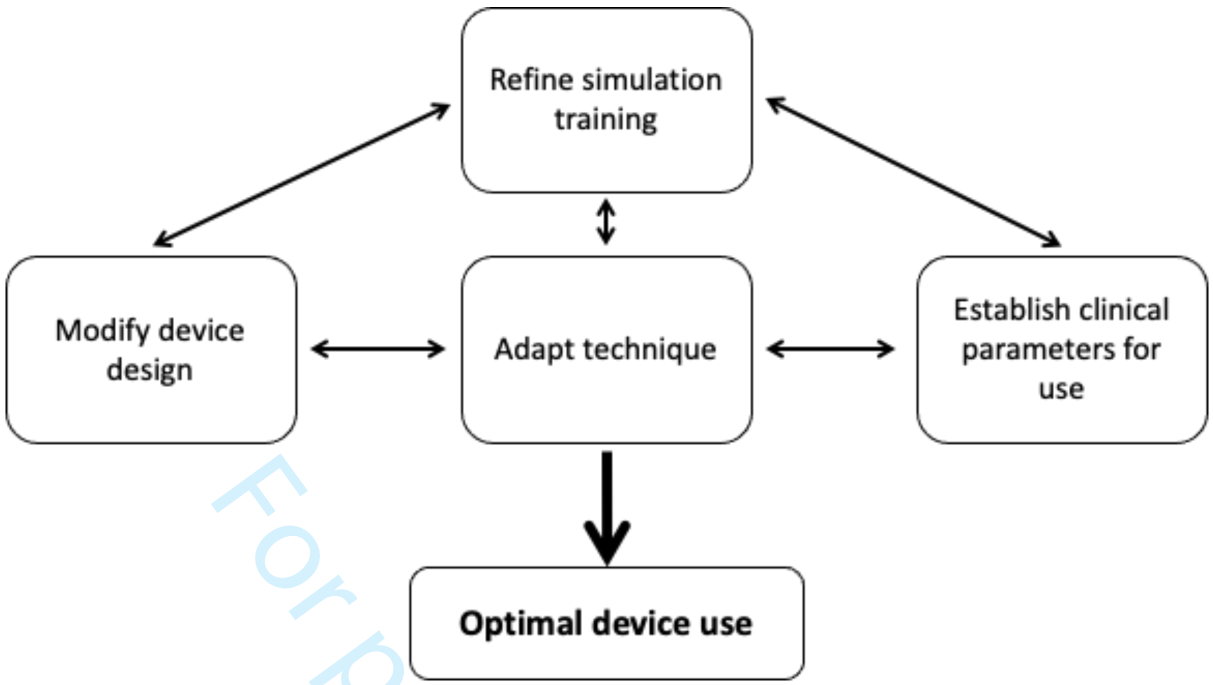

For peer review only

REVISION RECORD

REV (VER)	REVISION DESCRIPTION	CR NO.
01(A)	INITIAL RELEASE	CM500000111539

BD Odon Device™

Inflatable device for assisted vaginal birth – Instructions For Use

Device Description

The **BD Odon Device™** is an instrument for assisted vaginal birth. The device uses a polyethylene sleeve and an inflatable air cuff to help ease the fetal head out of the birth canal. The BD Odon Device consists of an applicator, sleeve and fastening band.

Indications for Use

The BD Odon Device is envisaged to perform assisted vaginal birth. The main indications for the BD Odon Device are as follows:

- Prolonged second stage of labour
- Suspected fetal compromise in the second stage of labour
- Other medical indications to shorten second stage of labour

Contraindications

The use of the BD Odon Device is contraindicated in the following conditions:

- Cervix is not fully dilated
- Cephalopelvic disproportion/ irreducible moulding and large caput indicative of cephalopelvic disproportion
- Fetal head not fully engaged
- Undefined presentation
- Breech, transverse, face, brow presentations
- Membranes are not ruptured
- Contraindications for vaginal deliveries
- Untrained operator

General Warnings

1. The BD Odon Device should be used by skilled birth attendants who are:
   a. Trained to recognize the conditions for safe and effective application of the BD Odon Device:
      - Full cervical dilation
      - Fetal head position defined
      - Fetal head station defined

   b. Trained to use the BD Odon Device
2. The BD Odon Device is intended for single use. Do not reuse. Device function may be impaired through reuse or cleaning. Degradation of materials or failure to correctly reassemble the device could result in harm for the mother and the fetus. Reusing the product after exposure to biological materials may cause adverse patient reactions due to contamination.
3. As with other devices for assisted vaginal birth, the BD Odon Device could fail to deliver the baby.
   The criteria to define failure and to abandon the procedure are:
   a. No progression in descent of the fetal head after a total of three pulls
   b. No more than two attempts at application (no more than two device slippages)
   c. Duration of procedure longer than 20 minutes
4. As with other instruments for assisted vaginal birth, use of the BD Odon Device may result in maternal and neonatal complications. The most common complications with the use of forceps and ventouse include maternal trauma, neonatal facial injury (forceps), cephalohaematoma, subgaleal haemorrhage, and retinal haemorrhage (ventouse).
   Presently human, animal and simulated studies have not identified potential complications specific to the BD Odon Device therefore this section will be updated after analyses of the safety outcomes of the proposed randomised clinical study.
5. This BD Odon Device is exclusively for clinical investigation only.
6. Ensure packaging is not damaged prior to use. Discard product if packaging is found to be damaged.

General Precautions

As with other devices for assisted vaginal birth the following precautions should be taken before applying the BD Odon Device to ensuring that conditions for safe application of device are met:

- Verify full dilation of cervix, fetal head station at the ischial spines or below
- Verify cephalic vertex presentation (Occiput anterior, Occiput posterior, Occiput transverse positions)
- Verify rupture of membranes
- Provide adequate analgesia according to facility procedures
- Position women in lithotomy position
- Empty bladder
- Re-confirm fetal position
- Lubricate the birth canal

Symbol Legends

STERILE R	Sterilized using radiation
	Do not re-use
	Do not re-sterilize
	Do not use if package is damaged
	Fragile, handle with care
	Keep away from sunlight
	Keep dry
LATEX	Contains or presence of natural rubber latex
LOT	Batch code
	Use-by date
	Date of manufacture
	Manufacturer
EC REP	Authorized representative in the European Community
i	Consult instructions for use
	Caution
SN	Serial number

Manufacturer: Becton Dickinson Medical Products Pte Ltd, 30 Tuas Avenue 2, Singapore 639461

EC REP Authorised representative in the European Community:
Becton Dickinson France SAS, 11 rue Aristide Bergès, ZI des Iles, 38800 Le Pont de Claix, France

Odon Device is a trademark of Air Bag One. BD, the BD Logo, and all other trademarks are property of Becton, Dickinson and Company. © 2017 BD

For peer review only

VENDOR INFO:

-HOLDING LINES ONLY... DO NOT PRINT.
NOT USE FOR DIMENSIONAL PURPOSES
-IFU DIMENSIONS TO BE 210MM X 297MM.

PMS2755

BD

BDMP R&D
30 TUAS AVENUE 2 S(639461)
SINGAPORE

THIS MATERIAL IS THE CONFIDENTIAL AND PROPRIETARY PROPERTY OF BECTON DICKINSON AND COMPANY. NO PORTION MAY BE USED, REPRODUCED OR DISTRIBUTED WITHOUT PRIOR WRITTEN PERMISSION.

REV (VER)	01(A)
CR NO.	CM500000111539
REVISION DATE	-
TITLE	BD Odon Device™ - Instruction For Use
DESIGNED BY	Narayanan V
DRAFTED BY	Narayanan V
CREATION DATE	24 NOV 2017
FILE NAME	SRD-DGP0036_rev 01
UNITS	METRIC/INCHES UNLESS OTHERWISE SPECIFIED
SCALE	Scale Size 1:1 NOT PRINTED TO SCALE
SHEET	1 OF 2
TYPE	EXPERIMENTAL
DRAWING NUMBER	SRD-DGP0036

Document: SRD-DGP0036 Doc Type: ZLB Status: Released EFFECTIVE
Valid From: 07-Dec-2017 To: 31-Dec-9999 Doc Part: EN Revision: 01 Change #: 500000111539
Print Date: 07-Dec-2017 05:24:29 GMT Standard Time Usage: Prototype Usage Version: A Classification: Restricted

DEVICE USE STEPS (FOR CLINICAL INVESTIGATION ONLY)

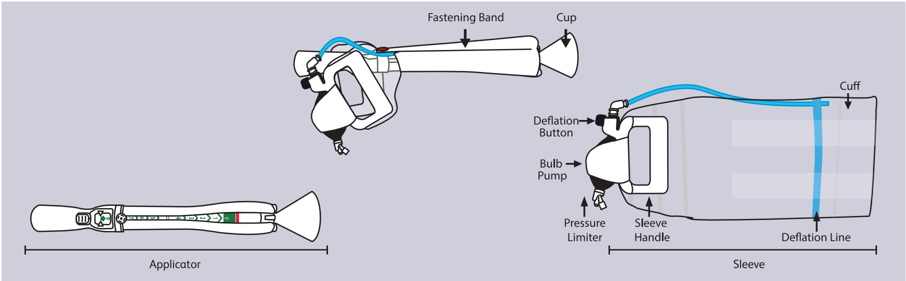

1. Ensure conditions for safe application of device are met:
   a. Full dilation of cervix, fetal head 0 to + 3 station, cephalic vertex presentation (OA, OP, OT positions), rupture of membranes
   b. Provide adequate analgesia according to facility procedures
   c. Position women in lithotomy position
   d. Empty bladder
   e. Re-confirm fetal position
   f. Lubricate the birth canal

2. Remove BD Odon Device from packaging without compromising the sterility of the device.

3. Pull back the fastening band until the blue deflation line is exposed.

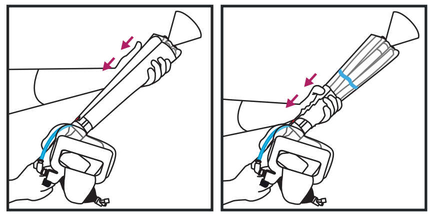

4. With the fastening band still intact, generously lubricate the inside and outside of the sleeve and the cup.

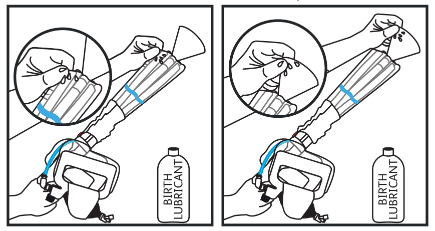

5. While holding the applicator handle gently slide the fastening band back to the top of the sleeve.

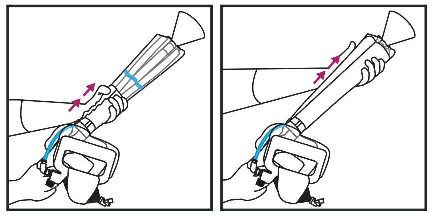

6. Grip the applicator handle and ensure the viewing window is facing upwards.

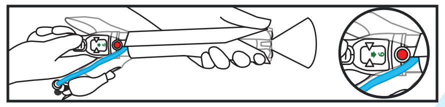

7. Fold the cup and gently insert it through the vulva and check it has regained its circular shape.

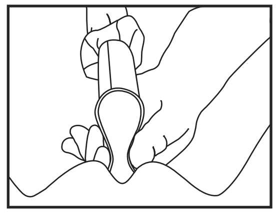

8. Check that there is no maternal tissue trapped between the cup and the fetal head.

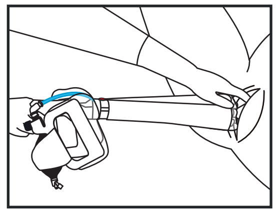

9. With your hand on the handle of the applicator, gently push the device through the vulva. Use your other hand to guide the device and do not apply any force with it.

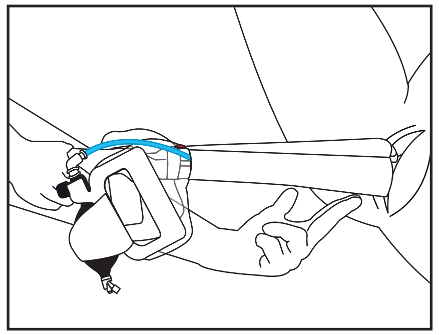

A

DEVICE USE STEPS (FOR CLINICAL INVESTIGATION ONLY)

10. Unfasten the red button

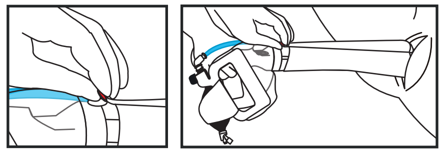

11. Open and completely remove the fastening band.

Note: ensure the sleeve and applicator remain in place inside the vulva.

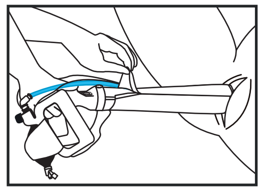

12. Between contractions, keeping both hands away from the sleeve, continue to gently push the applicator, starting at 45° below the horizontal and following the curvature of the birth canal. Monitor progress by looking through the viewing window.

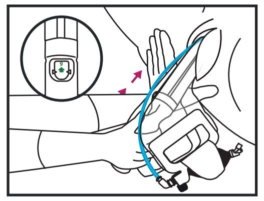

13. Continue to insert the device and stop when "0" appears in the viewing window.

Note: device is fully inserted when "0" appears in the viewing window.

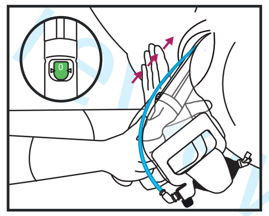

14. Squeeze the bulb pump fully and firmly at least 8 times to inflate the cuff.

Note: There is a pressure limiter in the bulb which prevents over inflation of the cuff.

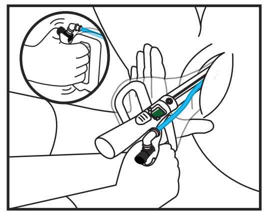

15. While protecting the perineum with one hand, use the other hand to completely withdraw the applicator and cup, leaving only the sleeve in place.

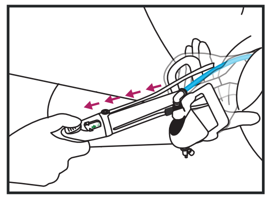

16. To compensate for possible reduction in cuff pressure, squeeze the bulb pump fully and firmly 2 more times prior to traction.

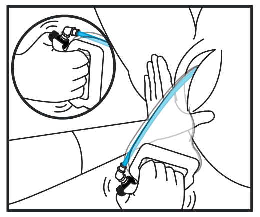

17. Grasp the sleeve handle, and during contractions pull gently and progressively following the J-shape of the birth canal.

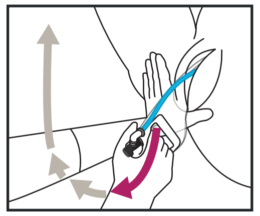

18. While continuing to pull gently along the J-shape of the birth canal, confirm the fetal head is descending with pulling efforts.

Note: if baby is not delivered at the first contraction repeat steps 16, 17 and 18 with any subsequent contractions.

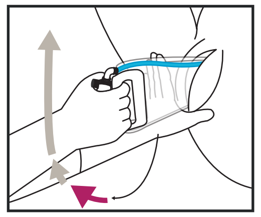

19. Once you see the blue deflation line completely (approximately at crowning) deflate the cuff by pushing on the blue deflation button. Pull the sleeve handle continuing to press the deflation button following the J-shape of the birth canal.

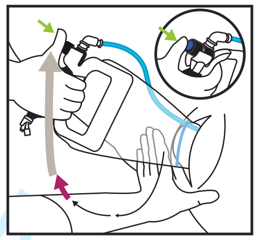

20. Continue to pull the sleeve handle while pressing the blue deflation button to pull the fetal head until the sleeve detaches from the head.

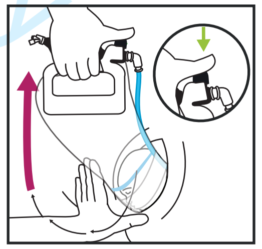

21. Proceed to assist the birth of the baby as per normal procedure.

22. Discard disposable applicator and sleeve according to local appropriate procedure. Do not reuse.

B

For peer review only

BD

BDMP R&D
30 TUAS AVENUE 2 S(639461)
SINGAPORE

THIS MATERIAL IS THE CONFIDENTIAL AND PROPRIETARY PROPERTY OF BECTON DICKINSON AND COMPANY. NO PORTION MAY BE USED, REPRODUCED OR DISTRIBUTED WITHOUT PRIOR WRITTEN PERMISSION.

REV (VER)	01(A)
CR NO.	CM500000111539
REVISION DATE	-
TITLE	BD Odon Device™ - Instruction For Use
DESIGNED BY	Narayanan V
DRAFTED BY	Narayanan V
CREATION DATE	24 NOV 2017
UNITS	METRIC/INCHES UNLESS OTHERWISE SPECIFIED
SCALE	Scale Size 1:1 NOT PRINTED TO SCALE
SHEET	2 OF 2
TYPE	EXPERIMENTAL
DRAWING NUMBER	SRD-DGP0036
FILE NAME	SRD-DGP0036_rev 01

VENDOR INFO:

-HOLDING LINES ONLY... DO NOT PRINT.
NOT USE FOR DIMENSIONAL PURPOSES
-IFU DIMENSIONS TO BE 210MM X 297MM.

PMS2755

For peer review only - http://bmjopen.bmj.com/site/about/guidelines.xhtml

Document: SRD-DGP0036 Doc Type: ZLB Status: Released EFFECTIVE
Valid From: 07-Dec-2017 To: 31-Dec-9999 Doc Part: EN Revision: 01 Change #: 500000111539
Print Date: 07-Dec-2017 05:24:29 GMT Standard Time Usage: Prototype Usage Version: A Classification: Restricted

Table S3 Standards for Reporting Qualitative Research (SRQR)

No.	Topic	Page number in manuscript
	**Title and abstract**	
S1	Title	1
S2	Abstract	2-3
	**Introduction**	
S3	Problem formulation	5-8
S4	Purpose or research question	5
	**Methods**	
S5	Qualitative approach and research paradigm	5-6
S6	Research characteristics and reflexivity	5-7
S7	Context	5-6
S8	Sampling strategy	6
S9	Ethical issues pertaining to human subjects	18
S10	Data collection methods	7
S11	Data collection instruments and technologies	7
S12	Units of study	7
S13	Data processing	7-8
S14	Data analysis	7-8
S15	Techniques to enhance trustworthiness	6-8
	**Results/findings**	
S16	Synthesis and interpretation	8-13
S17	Links to empirical data	8-13
	**Discussion**	
S18	Integration with prior work, implications, transferability, and contribution(s) to the field	14-17
S19	Limitations	14-17
	**Other**	
S20	Conflicts of interest	17
S21	Funding	8

For peer review only

No.	Topic	Item
	**Title and abstract**	
S1	Title	Concise description of the nature and topic of the study Identifying the study as qualitative or indicating the approach (e.g., ethnography, grounded theory) or data collection methods (e.g., interview, focus group) is recommended
S2	Abstract	Summary of key elements of the study using the abstract format of the intended publication; typically includes background, purpose, methods, results, and conclusions
	**Introduction**	
S3	Problem formulation	Description and significance of the problem/phenomenon studied; review of relevant theory and empirical work; problem statement
S4	Purpose or research question	Purpose of the study and specific objectives or questions
	**Methods**	
S5	Qualitative approach and research paradigm	Qualitative approach (e.g., ethnography, grounded theory, case study, phenomenology, narrative research) and guiding theory if appropriate; identifying the research paradigm (e.g., postpositivist, constructivist/ interpretivist) is also recommended; rationale[b]
S6	Researcher characteristics and reflexivity	Researchers' characteristics that may influence the research, including personal attributes, qualifications/experience, relationship with participants, assumptions, and/or presuppositions; potential or actual interaction between researchers' characteristics and the research questions, approach, methods, results, and/or transferability
S7	Context	Setting/site and salient contextual factors; rationale[b]
S8	Sampling strategy	How and why research participants, documents, or events were selected; criteria for deciding when no further sampling was necessary (e.g., sampling saturation); rationale[b]
S9	Ethical issues pertaining to human subjects	Documentation of approval by an appropriate ethics review board and participant consent, or explanation for lack thereof; other confidentiality and data security issues
S10	Data collection methods	Types of data collected; details of data collection procedures including (as appropriate) start and stop dates of data collection and analysis, iterative process, triangulation of sources/methods, and modification of procedures in response to evolving study findings; rationale[b]
S11	Data collection instruments and technologies	Description of instruments (e.g., interview guides, questionnaires) and devices (e.g., audio recorders) used for data collection; if/how the instrument(s) changed over the course of the study
S12	Units of study	Number and relevant characteristics of participants, documents, or events included in the study; level of participation (could be reported in results)
S13	Data processing	Methods for processing data prior to and during analysis, including transcription, data entry, data management and security, verification of data integrity, data coding, and anonymization/deidentification of excerpts
S14	Data analysis	Process by which inferences, themes, etc., were identified and developed, including the researchers involved in data analysis; usually references a specific paradigm or approach; rationale [b]
S15	Techniques to enhance trustworthiness	Techniques to enhance trustworthiness and credibility of data analysis (e.g., member checking, audit trail, triangulation); rationale[b]
	**Results/findings**	
S16	Synthesis and interpretation	Main findings (e.g., interpretations, inferences, and themes); might include development of a theory or model, or integration with prior research or theory
S17	Links to empirical data	Evidence (e.g., quotes, field notes, text excerpts, photographs) to substantiate analytic findings
	**Discussion**	
S18	Integration with prior work, implications, transferability, and contribution(s) to the field	Short summary of main findings; explanation of how findings and conclusions connect to, support, elaborate on, or challenge conclusions of earlier scholarship; discussion of scope of application/ generalizability; identification of unique contribution(s) to scholarship in a discipline or field
S19	Limitations	Trustworthiness and limitations of findings
	**Other**	
S20	Conflicts of interest	Potential sources of influence or perceived influence on study conduct and conclusions; how these were managed
S21	Funding	Sources of funding and other support; role of funders in data collection, interpretation, and reporting

[a]The authors created the SRQR by searching the literature to identify guidelines, reporting standards, and critical appraisal criteria for qualitative research; reviewing the reference lists of retrieved sources; and contacting experts to gain feedback. The SRQR aims to improve the transparency of all aspects of qualitative research by providing clear standards for reporting qualitative research.

[b]The rationale should briefly discuss the justification for choosing that theory, approach, method, or technique rather than other options available, the assumptions and limitations implicit in those choices, and how those choices influence study conclusions and transferability. As appropriate, the rationale for several items might be discussed together.

ACADEMIC MEDICINE

only

BMJ Open

Novel device for assisted vaginal birth: using integrated qualitative case study methodology to optimise Odon Device use within a feasibility study in a maternity unit in the Southwest of England

Journal:	*BMJ Open*
Manuscript ID	bmjopen-2021-059115.R1
Article Type:	Original research
Date Submitted by the Author:	12-Jun-2022
Complete List of Authors:	Hotton, Emily; University of Bristol; North Bristol NHS Trust<br>Blencowe, Natalie; University of Bristol, Centre for Surgical Research, School of Social and Community Medicine<br>Bale, Nichola; North Bristol NHS Trust<br>Lenguerrand, Erik; University of Bristol School of Clinical Science, School of Clinical Sciences, Musculoskeletal Research Unit<br>Draycott, Tim ; North Bristol NHS Trust<br>Crofts, Joanna; North Bristol NHS Trust<br>Wade, Julia; University of Bristol, Bristol Medical School
**Primary Subject Heading**:	Obstetrics and gynaecology
Secondary Subject Heading:	Qualitative research
Keywords:	QUALITATIVE RESEARCH, OBSTETRICS, STATISTICS & RESEARCH METHODS
Note: The following files were submitted by the author for peer review, but cannot be converted to PDF. You must view these files (e.g. movies) online.	
S2 Video.MOV	

SCHOLARONE™
Manuscripts

I, the Submitting Author has the right to grant and does grant on behalf of all authors of the Work (as defined in the below author licence), an exclusive licence and/or a non-exclusive licence for contributions from authors who are: i) UK Crown employees; ii) where BMJ has agreed a CC-BY licence shall apply, and/or iii) in accordance with the terms applicable for US Federal Government officers or employees acting as part of their official duties; on a worldwide, perpetual, irrevocable, royalty-free basis to BMJ Publishing Group Ltd ("BMJ") its licensees and where the relevant Journal is co-owned by BMJ to the co-owners of the Journal, to publish the Work in this journal and any other BMJ products and to exploit all rights, as set out in our licence.

The Submitting Author accepts and understands that any supply made under these terms is made by BMJ to the Submitting Author unless you are acting as an employee on behalf of your employer or a postgraduate student of an affiliated institution which is paying any applicable article publishing charge ("APC") for Open Access articles. Where the Submitting Author wishes to make the Work available on an Open Access basis (and intends to pay the relevant APC), the terms of reuse of such Open Access shall be governed by a Creative Commons licence – details of these licences and which Creative Commons licence will apply to this Work are set out in our licence referred to above.

Other than as permitted in any relevant BMJ Author's Self Archiving Policies, I confirm this Work has not been accepted for publication elsewhere, is not being considered for publication elsewhere and does not duplicate material already published. I confirm all authors consent to publication of this Work and authorise the granting of this licence.

For peer review only

Novel device for assisted vaginal birth: using integrated qualitative case study methodology to optimise Odon Device use within a feasibility study in a maternity unit in the Southwest of England

Emily J Hotton[1,2] *, Natalie S Blencowe[3,4], Nichola Bale[2], Erik Lenguerrand[1,2], Tim J Draycott[2], Joanna F Crofts[2], Julia Wade[5]

1 Translational Health Sciences, University of Bristol, Bristol, UK

2 Southmead Hospital, North Bristol NHS Trust, Bristol, UK

3 University Hospitals Bristol NHS Foundation Trust, Bristol, UK

4 Centre for Surgical Research, Population Health Sciences, University of Bristol, Bristol, UK

5 Population Health Sciences, University of Bristol, Bristol, UK

*** Corresponding Author:**

Women and Children's Academic Research

The Chiltern's

Maternity Unit

Southmead Hospital

Bristol

United Kingdom

BS10 5NB

Tel: 0117 414 6764

Email: emily.hotton@nhs.net

Running title: Optimising the use of the Odon Device

Abstract word count: 300

Word count: 3939

For peer review only - http://bmjopen.bmj.com/site/about/guidelines.xhtml

Objective

When novel devices are used 'in human' for the first time, their optimal use is uncertain because clinicians only have experience from pre-clinical studies. This study aimed to investigate factors that might optimise use of the Odon Device for assisted vaginal birth.

Design

We undertook qualitative case studies within the ASSIST Study, a feasibility study of the Odon Device. Each 'case' was defined as one use of the device and included at least one of the following: observation of the attempted assisted birth, and an interview with the obstetrician, midwife or woman. Data collection and thematic analysis ran iteratively and in parallel.

Setting

Tertiary referral NHS maternity unit in the Southwest of England.

Participants

Women requiring a clinically indicated assisted vaginal birth.

Intervention

The Odon Device, an innovative device for assisted vaginal birth.

Primary and secondary outcomes measures

Determining the optimal device technique, device design and defining clinical parameters for use.

Results

Thirty-nine cases involving an attempted Odon assisted birth were included in this study, of which 19 resulted in a successful birth with the device. Factors that improved use included optimisation of device technique, device design and clinical parameters for use. Technique adaptations included: applying the device during, rather than between, contractions; having a flexible approach to the application angle; and deflating the air cuff sooner than originally

For peer review only

For peer review only - http://bmjopen.bmj.com/site/about/guidelines.xhtml

proposed. Three design modifications were proposed involving the deflation button and sleeve. Although use of the device was found to be appropriate in all fetal positions, it was considered contraindicated when the fetal station was at the ischial spines.

Conclusions

Case study methodology facilitated the acquisition of rapid insights into device function in clinical practice, providing key insights regarding use, design, and key clinical parameters for success. This methodology should be considered whenever innovative devices are introduced into clinical practice.

Trial registration

ASSIST Study registration: ISRCTN10203171 https://doi.org/10.1186/ISRCTN10203171

Keywords

Assisted vaginal delivery, qualitative research, translational research, Medical Devices

For peer review only

Article Summary

- Case study methodology including data from participant observation and/or interviews (with operators, midwives and/or women), was successfully used in an intrapartum setting to evaluate the use of a novel device, the Odon Device, for assisted vaginal birth.
- Iterative data analysis and feedback of findings enabled rapid dissemination of findings to key stakeholders, and consensus regarding future alterations to device design, technique, and selection criteria for optimal device use.
- Observations were undertaken where possible; however, due to the unpredictable nature of AVBs it was not possible to attend them all, potentially impacting on the generalisability of our findings.

For peer review only

For peer review only - http://bmjopen.bmj.com/site/about/guidelines.xhtml

Introduction

Each year approximately 82,000 women in the UK have an assisted vaginal birth (AVB).[1] In recent years, despite the known advantages of AVB, this rate has reduced with a corresponding increase in Caesarean births in the second stage of labour. Current devices for AVB require a high level of training and skill (with additional expertise required to define the fetal position) and can be associated with significant maternal and neonatal morbidity if used incorrectly.[2] An innovative device that is easier and safer to use, could increase women's access to AVB, which in turn would help to reduce the number of emergency Caesarean sections performed in the second stage.[3,4]

Before introducing devices into widespread practice, it is necessary to evaluate their safety and efficacy, and obtain CE marking. However, other factors – such as device technique, design, and clinical parameters for use – are not routinely assessed, yet may ultimately limit their success. Preliminary feasibility work exploring these other factors may be valuable prior to evaluation within a definitive randomised controlled trial. The Odon Device (**Figure 3**) has undergone rigorous pre-clinical[5], simulation[6,7], human factors[8] and Phase 1 first-in human investigation[9] which conclude that it appeared to be safe. However, the Odon Device has hitherto not been used in the intended population: women requiring an AVB. The Odon Device was originally designed by Jorge Odón and has since been developed by a team of clinicans and medical engineers. It assisted birth using an inflatable air cuff attached to handles (Figure 1).

This study applied qualitative case study methodology to examine in detail how the Odon Device (version 4.1) is used for AVB, and to determine what factors may impact on optimal use. The study was embedded in the ASSIST Study – a feasibility study of the Odon Device.[10,11]

Figure 1 Diagram of the Odon Device

Methods

Research design

For peer review only

The ASSIST Study[10,11] was conducted in a maternity unit in the Southwest of England with full detail published elsewhere.[11,12] Integrated within the study was qualitative research, using case study methodology to explore the factors that may influence optimum device use. Case study methodology is particularly suited to answering 'how and why' questions and providing in-depth contextual detail, essential in early evaluations[13] of complex interventions,[14–16] such as use of a novel device for AVB,[17] and has previously been used to explore surgical innovation.[18] In this study, each 'case' was defined as one use of the device and included at least one of the following: observation of the attempted Odon assisted birth and/or an interview with the obstetrician, midwife or woman. The researcher ensured that the use of the device in the study was compared against the Instructions For Use (IFU) document, which is mandated by regulatory bodies as one of the processes to ensure device safety and efficacy. Given the focus of this paper is of the technical aspects of device use, data presented reports observation and healthcare professional interview findings. Data reporting experiences of women are presented separately.[12]

Participants

There were two groups of participants for the case studies: women and healthcare professionals (obstetricians and midwives). All women participating in the ASSIST Study were eligible to be included in the case study research and gave written consent.[12] All trained operators and midwives provided written consent. There were five operators, three consultants and two registrars.

Sampling

Typical sampling for observation was in part purposive and in part opportunistic (i.e. dependent on the researcher being on site and available to conduct the observation). The aim was to include a range of clinical indications for AVB and a range of operators.

For peer review only

134 **Patient and public involvement**

135 Patients and the public were involved in all aspects of the ASSIST Study, as previously reported.[10,12]

137

138 **Data collection**

139 Included case studies comprised data from one or more of the following sources: observations of the AVBs and/or interviews with women, midwives and operators. Observations, including technical details, contextual factors and communication, were prospectively recorded on a bespoke observation schedule. Detailed observations of the operative steps performed by the obstetrician during AVBs were recorded enabling a stepwise account of the 'usual steps' to be generated and compared against the IFU. The original IFU were developed prior to the ASSIST study during phase 1 clinical and simulation studies and included 22 operative steps.[8,9] In these IFU, the AVB was divided into six domains according to purpose (**Table 1**). The IFU and instructional video for operators used for the Odon Device in the ASSIST Study can be viewed in Supplementary files 1 and 2.

149 All women who had the birth of their baby formally observed were invited to participate in an interview at day one postnatal and clinicians within five days following the assisted birth.[10] In line with usual practice in conducting case study research a flexible approach was taken to which data were collected for each case, based on the value of insights gained for each data source. Any method of data collection (observation or interview) could be suspended if it was observed to be delivering no new insights.

155

156 **Data analysis**

157 Data collection and analysis were iterative and ran in parallel using the six-step framework described for thematic analysis.[19] Data analysis was largely inductive although some deduction derived from using the IFU as a framework against which to evaluate what took place. All data for each case were read together to identify and organise codes. Codes were

For peer review only

developed using text that captured significant views in the data, then grouped to reflect developing themes, with code descriptions and sample quotes assigned. Double coding of a proportion of interview transcripts (20%) was undertaken by JW. A narrative report was created for each case, triangulating all available data. Any issues requiring clarification were highlighted during the creation of the report, for exploration during subsequent interviews. Commonality and variances across cases were discussed between the researchers and used to further shape evolving themes and sampling. This systematic analysis supported rapid within- and cross-case comparison. Nvivo 12 (QSR International, Melbourne, Australia) was used to organise data and support analysis.

Feedback of findings

Iterative data collection and analysis enabled the rapid identification of key learning points or corrections to technique for dissemination to operators (see Table 2). Key findings were relayed rapidly to operators using messages via an end-to-end encryption platform, regular face-to-face discussions and operator debriefs. Furthermore, following the 36th Odon assisted birth, an interactive summit was held with key stakeholders (the clinical research team, design engineers, statisticians, and funders), with the aim of sharing learning experiences and gaining consensus regarding any changes that may be suggested.

Funding

This work was supported by the Bill & Melinda Gates Foundation [INV-010180]. Under the grant conditions of the Foundation, a Creative Commons Attribution 4.0 Generic License has already been assigned to the Author Accepted Manuscript version that might arise from this submission.

Results

Forty births were assisted with the Odon Device at North Bristol NHS Trust, UK, between October 2018 and January 2019. One case had no qualitative data because the researcher

For peer review only - http://bmjopen.bmj.com/site/about/guidelines.xhtml

was unavailable, resulting in 39 case studies arising from 40 (97.5%) single uses of the Odon Device (**Table 2**). Data for the cases studies included eight observations and accompanying interviews with the women, 19 midwife interviews, 37 operator interviews and two operator reflections (Table 2). All births were assisted in the lithotomy position. Ninety percent of women had a perineal tear, including 28 episiotomies and three women (8%) sustained a third-degree perineal tear.[10] Nineteen births were successfully assisted with the Odon Device. Of those that were unsuccessful, 19 were assisted by forceps and two by Caesarean section. There were no serious maternal or neonatal adverse events related to the use of the device and there were no serious adverse device effects. Four devices (10%) were ineffective due to a manufacturing fault.[10] Observations varied in length from 33 to 68 minutes. Interviews with women lasted 6.5 to 9.6 minutes, interviews with operators lasted between 5.4 and 26.1 minutes and interviews with midwives lasted 3.4 to 13.2 minutes. The shorter interviews with operators and midwives were all from cases in which the Odon Device was used successfully. Interviews for cases in which the Odon Device was unsuccessful were often longer as there were more aspects of device use to discuss. Another potential reason some interviews were short is that all operators and midwives were interviewed more than once, meaning they often did not have additional comments in subsequent interviews.

It became apparent that there were three factors contributing to optimisation of device use: (i) device technique, (ii) device design and (iii) clinical parameters for device use (Figure 2).

Figure 2 How case study methodology may be able to determine optimal device use through bridging multiple factors relating to the device

Device technique

Suggested adaptions to the original IFU included (i) device application during rather than between contractions, (ii) altering the application angle and (iii) deflating the air cuff as soon as any aspect of the blue deflation line became visible.

Device application with a contraction

For peer review only - http://bmjopen.bmj.com/site/about/guidelines.xhtml

The original IFU stated that the Odon Device should be applied between contractions, as was standard practice with forceps and ventouse. It became apparent during the first two attempted AVBs that this disimpacted the fetal head out of the pelvis and operators were unable to correctly place the device:

> D1: *'Again, I had to use significant pressure to try and get the device over the fetal head. And loads of liquor came down during the application suggesting that there was some degree of disimpaction.'*

By the third attempted birth, operators had adapted their technique to include fundal pressure to aid application, which resulted in successful device application and the first successful AVB. The use of fundal pressure, although successful, was not well tolerated by women without a regional anaesthetic:

> M3: *'Significant fundal pressure that was used at the time…she was uncomfortable…maybe that will be something up for review.'*

Following feedback from qualitative findings, the application technique was adapted again during the eighth birth. This was the first time the Odon Device was applied during a contraction without the use of fundal pressure, resulting in a successful application and birth. Fundal pressure was only used in a small number of births and quickly dropped from the technique as soon as application with a contraction was found to be successful:

> D2: *'I haven't used fundal pressure since delivery number two or three for me, but what has been very successful is putting it on during a contraction. I think.'*

Device application angle

The original IFU stated that the device should be applied 'starting at 45° below the horizontal'. By the eighth attempted birth it was apparent that this was not optimal and operators naturally moved to a more 'horizontal' application:

> D2: *'I definitely pushed the device in at a much flatter angle, much more parallel with the bed than I had in the past...'*

For peer review only - http://bmjopen.bmj.com/site/about/guidelines.xhtml

All operators quickly agreed that the angle required might be dependent on factors such as fetal position and station:

> D1: '*So I was kind of like, "Oh, OP, it might be more, you know, it could be difficult because it's an OP…"*'

> D2: *'I think we've still got to continue experimenting or changing the angle of insertion. I think there may be an optimum angle of insertion or it may be that we have to change angle of insertion for different stations...'*

Deflating the device

The original IFU stated that 'once you see the blue deflation line completely' the air cuff should be deflated. By the third attempted birth it became apparent to the observer that this was too late and that the optimum time for air cuff deflation seemed to be when any section of the blue line could be seen:

> O5: '*Noticed that it was not the anterior blue deflation line that the operator was looking at the deflate but the posterior deflation line, due to the fact that there is an acute J curve and anterior line not seen. Will need to change this in training.'*

These observations were fed back rapidly and iteratively to the Odon Device operators and further discussed with the wider research team at the Odon Summit (Table 2).

Device design and performance

Multiple potential device adaptations were noted during the case study research. Four design modifications for future device adaptations were identified: (i) strengthening the sleeve seal lines, (ii) creating a wider opening between the sleeve handles, (iii) altering the design of the deflation button and (iv) address the manufacturing fault that was identified.

Sleeve seal lines and opening between sleeve handles

One operator noted that the sleeve seal lines tore during traction, on several occasions:

For peer review only

D4: *'… the sleeve is not sturdy… it might actually rip it open, which has happened with me a few times.'*

During device inspection, it was noted that all devices had small tears (<2cm) in the seal lines of the sleeve, and one had a significant tear (>7cm). There was no evidence any of these tears had had a negative effect on the function of the Odon Device, indeed the device with a significant tear achieved a successful Odon birth. Tearing was thought to have occurred when operators opened the sleeve handles before and between tractions to physically look at the station of the vertex. In contrast to standard devices used for AVB, there was little proprioceptive feedback to ascertain the station of the baby, so visual inspection was useful:

O5: *'…I got the impression that the operator was unsure as to whether the head had descended so opened the handles to look inside the sleeve.'*

Following interviews, it was suggested that the opening between the two handles was made wider to enable operators to view the progression of the baby's head more easily (Figure S1). Ultrasound assessment was not used as this method was not routinely adopted in our unit at the time of the study.

Deflation button

In six cases it was noticed that the operator accidentally pressed the deflation button. Each time this occurred, the cuff was reinflated immediately. All operators agreed that the design of the deflation button should be altered to reduce the risk of inadvertent activation (Figure S2):

O3: '*Operator accidentally pressed the deflation button 'oh, whoops that was my fault, I'll just re-inflate'.*'

Manufacturing fault

For peer review only - http://bmjopen.bmj.com/site/about/guidelines.xhtml

All devices were disinfected and inspected following their use as per protocol.[10,11] During this inspection, four devices were found to have an ineffective bulb pump (**Error! Reference source not found.**) which resulted in inadequate cuff inflation (**Table 2**). Operators' comments during the attempted births reflected this, as the device did not act in the expected manner.

> D4: *'Yes, there was no grip… It just came out deflated, so it didn't feel right.'*

This prompted a rapid retrospective review of all used and stored devices to ensure that no other unsuccessful attempts were attributed to this fault, none were.

Optimal clinical parameters for Odon Device use

The Odon Device was used to successfully assist births in all fetal positions. Midwives particularly noted how the device could help deliver a baby in the occipito-posterior position which is a technically more challenging position:

> M9: '*I think, probably, it could be quite universal as an instrumental device. It didn't seem to matter whether the baby was OA* [occipito-anterior] *or* OP [occipito-posterior]...'

However, although the device could be successful at assisting birth in all positions, it became apparent that for women with fetal station at spines or a more complex presentation (such as brow or nuchal arm) the device was not successful. Operators were either unsuccessful at applying the device correctly onto the fetal head or the device simply slipped off the fetal head with the initial traction:

> *D1: 'So, it was direct OP at the spines, and it was almost coming to a brow, I could feel the orbital ridges…I was thinking, "Oh, I'm really not sure that this is going to work."…I didn't feel that was a failed Odon, that was a baby that was never going to come out vaginally.'* [unsuccessful Odon-failed rotational forceps, emergency Caesarean section]

For peer review only - http://bmjopen.bmj.com/site/about/guidelines.xhtml

As experience with the device increased, it became apparent that the device could be used comfortably without a regional anaesthetic (with only perineal infiltration of local anaesthetic). Device use was noted to be better tolerated than bladder emptying by urethral catheterisation, a procedure that is less invasive:

> D2: '*She actually found the catheterisation more uncomfortable than putting on the Odon Device with no analgesia at all.*'

Feedback to operators

All qualitative case study findings relating to device technique, design, and clinical parameters for use were presented to key stakeholders at the Odon Summit by the qualitative researcher. The case study research provided suggestions for device technique adaptation for some of the operative steps, but not for them all. It was agreed that there were still unanswered questions regarding the technique (such as which angle to use for application) and that further data was required to achieve this. Clinically important adaptations to device design were agreed upon (including altering the deflation button design) and the clinical parameters for use were confirmed, with an agreement that the device should not be used if the vertex is at the level of the ischial spines.

Discussion

Case study research identified three areas that could optimise device use: (i) device technique, (ii) device design and (iii) acceptable clinical parameters. Principal technique adaptations were centred on device application and deflation of the air cuff. The initial IFU specified a particular angle for device application however, during clinical use it became apparent that this angle needed to be flexible and was less acute than originally specified however, there was no consensus on the exact optimal angle and it was surmised that more data would be required to achieve this. Device modifications of altering the sleeve and deflation button were recommended for useability rather than to transform the functionality of the device. The manufacturing fault was quickly identified and rectified by the manufacturer through post-use device inspection. Optimal parameters for device use were proposed and focussed primarily on the station of the baby, with use at station spines recommended to be prohibited. Adaptations to optimise device use were adopted by the

For peer review only

manufacturer to create Odon Device (version 4.2) which was used in two further Odon Device feasibility studies, each studying 104 Odon assisted births. These have recently closed to recruitment in the UK[20] and France[21] and aimed to address the unanswered aspects of optimal device use, specifically the technique. These findings will be published once follow-up and data analysis in complete. Case study research enabled systematic, rapid generation of data and understanding of device use that enabled the researchers and manufacturers develop study protocols and device updates to support the ongoing investigation of the device.

Strengths and limitations

This was the first time that research has been undertaken on the Odon Device in clinically indicated cases and indeed the first-time case study research has been used to explore the use of devices for AVB. Device design and technique is unique to the device and although cannot be directly compared to other devices for AVB step-by-step, some comparisons and differences can be noted. The Odon Device, unlike other devices for AVB[22], can only be successfully applied during a contraction or with maternal effort, even though techniques for traction once the device is applied appear similar. Clinical indications for use are slightly different to that of forceps and ventouse in the UK.[22] In the UK, all currently used devices for AVB are permitted to be used at stations spines or below. We have demonstrated that this is not the case for the Odon Device, as we have demonstrated that this will not be successful. Interestingly, performing AVBs at station spines is not permitted in other countries.[23]

An AVB is a complex intervention, and this makes studying the use of the device challenging. Qualitative case study methodology has been used to explore technique in surgical procedures[18] however, there are no published examples of case study methodology being used to investigate novel devices. The case studies integrated participant observation as well as interviews with operators, midwives, and women to explore the introduction of an innovative device in context and in detail. The benefits of this were that experiences, and views of all stakeholders were easily obtained, and we were able to investigate operator views in detail. Triangulation of data linked to a particular case led to insights for amendments for optimum device use being identified more rapidly that if a single source of

For peer review only

qualitative data (e.g., observation or interview only) had been used. Rapid dissemination of findings resulted in prompt adoption of beneficial techniques for use. By using this methodology and incorporating data from all stakeholders (operators, midwives, and women) and observations we were able to gain a balanced and comprehensive assessment of the use of the device. When trying to understand optimal device use, operator interviews were found to be of crucial importance. Comparing case study data collected under different conditions (such as different analgesia, different presentations of babies, different operators) enabled commonalities and disparities in technique to be highlighted and thoroughly investigated. This enabled the clinical research team to propose evidence-based modifications to the device design and provide clarity on recommendations for clinical parameters for use. Case study methodology encouraged operators to reflect, critique and appraise their use of the device for each birth, resulting in enhanced and enriched communication between operators regarding their experiences through conversations and a dedicated operator messaging group. In future, data from encrypted social media platforms could be incorporated into the qualitative data for analysis. Reporting was undertaken following the Standards for Reporting Qualitative Research[24] (Supplementary information 3).

There are limitations to this study. The aim of understanding the optimal operative steps for device use, and thus confirming a finalised IFU were not met. For some operative steps consensus was reached as to the recommended course of action (such as applying the device with a contraction). However, for others more data were required (such as what specific angle of application to use). Case studies within the ASSIST Study were finite. Observations were undertaken where possible, however due to the unpredictable nature of AVBs it was not possible to attend all assisted births. Indeed, none of the more complex attempted AVBs performed in the operating theatre were observed. This could have an impact on the generalisability of the findings as births undertake in the operating theatre are often more technically challenging for operators. All interviews with clinicians were undertaken within five days following the assisted birth. Recollections of the clinicians may have been less accurate the longer the time between assisted birth and interview. The case studies were undertaken by a specialist trainee in obstetrics and gynaecology meaning that pre-conceptions and existing knowledge may have influenced the collection and

For peer review only - http://bmjopen.bmj.com/site/about/guidelines.xhtml

interpretation of the data, although at the time of commencing the case studies the researcher was naïve to the use of the Odon Device in the clinical setting. Lastly, operators may have changed their behaviours during observations, perhaps not reflecting their real-life practice.

There are two key next steps that should be considered. Firstly, feasibility of the use of the Odon Device for AVB should be undertaken in different healthcare settings. Thus far, research has been undertaken in high-income settings where AVB is used regularly. Exploring device using in low- and middle- income settings, including where rates of AVB are lower than the UK and France could help understand if there are further considerations for optimal device use that need to be addressed. Secondly, following the completion of the two further feasibility studies, a decision needs to be made as to whether the device is ready to be compared against available alternatives (forceps and ventouse) in a randomised controlled trial. As recommended by IDEAL-D[25] researchers need to be satisfied that the technique, design and clinical parameters for use are sufficiently stable to enable this to happen.

Conclusion

Case study methodology facilitated insights into optimal technique, design and clinical parameters for use of the Odon Device. Optimising use of a device is an essential prerequisite to evaluating outcomes, as it will impact directly on those outcomes and may result in lower-than-expected success rates. There were two clear factors that enhanced operator communication. Firstly, systematic triangulation of data from varying data sources provided a comprehensive, contextual overview of device use and rapid understanding of amendments required and secondly, rapid feedback of insights as they emerged to operators. This also facilitated operator consensus building, which was key in understanding and developing the iterative adaptations to the device technique, design and clinical parameters for device use. This is of paramount importance for getting operator buy-in for the next steps of device evaluation. This methodology should be considered whenever innovative devices are introduced to clinical trials and settings. It allows for rapid

For peer review only - http://bmjopen.bmj.com/site/about/guidelines.xhtml

assessment of device use and can support timely iterative adaptions to ensure there are minimal delays between device use in research and adoption in clinical practice.

Acknowledgements

The authors would like to thank all the women who agreed to take part in this research and all the maternity staff who enabled to safe completion of the ASSIST Study. The research was affiliated with but not funded by the NIHR Biomedical Research Centre at University Hospitals Bristol and Weston NHS Foundation Trust and the University of Bristol. The views expressed are those of the author(s) and not necessarily those of the NIHR or the Department of Health and Social Care, and the MRC ConDuCT-II (Collaboration and innovation for Difficult and Complex randomised controlled Trials In Invasive procedures) Hub for Trials Methodology Research (MR/K025643/1).

Competing interests

Authors report no competing interests.

Contribution to Authorship

EJH, NSB and JW developed the concept for the study. EJH performed all data collection and analysis with co-coding performed by JW. EJH wrote the initial draft of the manuscript, with support from NSB, JFC and JW. EJH, NSB, NB, EL, TJD, JFC and JW reviewed and approved the final manuscript.

Ethical approval

For peer review only

For peer review only - http://bmjopen.bmj.com/site/about/guidelines.xhtml

The research was approved by South Central–Berkshire REC, UK on 3rd September 2018 (18/SC/0344), the MHRA on 9th August 2018 and the HRA on 3rd September 2018.

Data sharing statement: Data are available upon reasonable request.

For peer review only

For peer review only - http://bmjopen.bmj.com/site/about/guidelines.xhtml

484 **Table/figure/video caption list**

485 **Table 1** Original components of application of the Odon Device for an assisted vaginal birth

486

487 **Table 2** Summary of 40 cases investigating the Odon Device with adaptations made to
488 device technique

489

490 **Figure 3** Diagram of the Odon Device

491

492 **Figure 4** How case study methodology may be able to determine optimal device use through
493 bridging multiple factors relating to the device

494

495 **Supplementary file (S1)** Odon Device IFU for Clinical Studies

496

497 **Supplementary file (S2)** Instructional video for the ASSIST Study
498 Video detailing how to use the Odon Device using a mannequin

499

500 **Supplementary file (S3)** Standards for Reporting Qualitative Research (SRQR)

501

502

For peer review only

For peer review only - http://bmjopen.bmj.com/site/about/guidelines.xhtml

References

1. NHS, Digital. NHS Maternity Statistics - England, 2013-14 [Internet]. 2015 [cited 2021 Oct 11]. Available from: http://digital.nhs.uk/catalogue/PUB16725

2. Hotton E, O'Brien S, Draycott TJ. Skills training for operative vaginal birth. Best Pract Res Clin Obstet Gynaecol. 2018;56:11–22.

3. Requejo JH, Belizán JM. Odon device: a promising tool to facilitate vaginal delivery and increase access to emergency care. Reprod Health. 2013;10:42–3.

4. Draycott TJ, Renzo GCD. The role of operative vaginal birth in the 21st century and a way forward. BJOG. 2017;124:5–6.

5. Lange C de, Saugstad OD, Solberg R. Assessment of cerebral perfusion with contrast-enhanced ultrasound during constriction of the neck mimicking malposition of the BD Odon DeviceTM: a study in newborn piglets. BJOG. 2017;124:26–34.

6. O'Brien SM, Winter C, Burden CA, Boulvain M, Draycott TJ, Crofts JF. Fetal head position and perineal distension associated with the use of the BD Odon DeviceTM in operative vaginal birth: a simulation study. BJOG. 2017;124:10–8.

7. O'Brien SM, Winter C, Burden CA, Boulvain M, Draycott TJ, Crofts JF. Pressure and traction on a model fetal head and neck associated with the use of forceps, KiwiTM ventouse and the BD Odon DeviceTM in operative vaginal birth: a simulation study. BJOG. 2017;124:19–25.

8. O'Brien SM, Mouser A, Odon JE, Winter C, Draycott TJ, Sumitro T, et al. Design and development of the BD Odon DeviceTM: a human factors evaluation process. BJOG. 2017;124:35–43.

9. Schvartzman JA, Krupitzki H, Merialdi M, Betrán AP, Requejo J, Nguyen MH, et al. Odon device for instrumental vaginal deliveries: results of a medical device pilot clinical study. Reprod Health. 2018;15:45–54.

10. Hotton EJ, Lenguerrand E, Alvarez M, O'Brien S, Draycott TJ, Crofts JF, et al. Outcomes of the novel Odon Device in indicated operative vaginal birth. Am J Obstet Gynecol. 2021;224:607:e1-17.

11. O'Brien S, Hotton EJ, Lenguerrand E, Winter C, Draycott TJ, Crofts JF, et al. The ASSIST Study - The BD Odon Device for assisted vaginal birth: a safety and feasibility study. Trials. 2019;20:159–67.

For peer review only

For peer review only - http://bmjopen.bmj.com/site/about/guidelines.xhtml

12. Hotton EJ, Blencowe NS, Lenguerrand E, Draycott TJ, Crofts JF, Wade J. The Odon Device to assist vaginal birth: Observations of and insights into women's experiences as narrated by health professionals and women. BMJ Open. 2021;11:e057023.

13. Yin RK. Case Study Research. 5th ed. CA: SAGE Publications, Inc; 2014.

14. Craig P, Dieppe P, Macintyre S, Michie S, Nazareth I, Petticrew M. Developing and evaluating complex interventions: The new Medical Research Council guidance. Int J Nurs Stud. 2013;50:587–92.

15. Campbell M, Fitzpatrick R, Haines A, Kinmonth AL, Sandercock P, Spiegelhalter D, et al. Framework for design and evaluation of complex interventions to improve health. BMJ. 2000;321:694.

16. Craig P, Dieppe P, Macintyre S, Michie S, Nazareth I, Petticrew M, et al. Developing and evaluating complex interventions: the new Medical Research Council guidance. BMJ. 2008;337:a1655-60.

17. Paparini S, Green J, Papoutsi C, Murdoch J, Petticrew M, Greenhalgh T, et al. Case study research for better evaluations of complex interventions: rationale and challenges. Bmc Med. 2020;18(1):301.

18. Blencowe NS, Blazeby JM, Donovan JL, Mills N. Novel ways to explore surgical interventions in randomised controlled trials: applying case study methodology in the operating theatre. Trials. 2015;16:589.

19. Braun V, Clarke V. Using thematic analysis in psychology. Qualitative Research in Psychology. 2006;3:77–101.

20. Hotton EJ, Alvarez M, Lenguerrand E, Wade J, Blencowe NS, Draycott TJ, et al. The Odon DeviceTM for assisted vaginal birth: a feasibility study to investigate safety and efficacy-The ASSIST II study. Pilot Feasibility Stud. 2021;7:72–81.

21. Mottet N, Bourtembourg A, Eckman-Lacroix A, Forner O, Mougey C, Metz JP, et al. [Focus on the Odon DeviceTM: Technical improvements, mechanical principles and progress of the clinical research program]. Gynecol Obstet Fertil Senol. 2020;48:814–9.

22. Murphy D, Strachan B, Bahl R, RCOG. Assisted Vaginal Birth: green-top guideline no.26. BJOG. 2020;127:e70–112.

23. ACOG. ACOG Practice Bulletin No. 154 Summary: Operative Vaginal Delivery. Obstet Gynecol. 2015;126:1118–9.

24. O'Brien BC, Harris IB, Beckman TJ, Reed DA, Cook DA. Standards for Reporting Qualitative Research: A Synthesis of Recommendations. Acad Med. 2014;89(9):1245–51.

25. Sedrakyan A, Campbell B, Merino JG, Kuntz R, Hirst A, McCulloch P. IDEAL-D: a rational framework for evaluating and regulating the use of medical devices. BMJ. 2016;353:i2372.

For peer review only - http://bmjopen.bmj.com/site/about/guidelines.xhtml

For peer review only

For peer review only - http://bmjopen.bmj.com/site/about/guidelines.xhtml

Table 1 Original components of application of the Odon Device for an assisted vaginal birth

Component	Steps within component
Preparation	Checking clinical pre-requisites for AVB<br>Lubricating the device
Device application	Removing the fastening band<br>Applying the device onto a fetal head
Cuff inflation	Ensuring the cuff is fully inflated in the correct position on the fetal head
Applicator removal	Removing the applicator from the fetal head
Traction	Following the J-shape curve of the pelvis applying traction with contractions
Removal of device	Deflating the air cuff as the fetal head is crowning

For peer review only

For peer review only - http://bmjopen.bmj.com/site/about/guidelines.xhtml

Table 2 Summary of 40 cases investigating the Odon Device with adaptations made to device technique

Case study no.	Successful (S) Unsuccessful (U) AVB with Odon and mode of birth	Observation	Interviews Women	Interviews Operator	Interviews Midwife	Device issues
1	U – Forceps	O1	W1	D1	M1	
2	U – Forceps			D2	M2	
Fundal pressure during device application tried						
3	S – Odon			D2	M2<br>M3	
Deflation of the air cuff when only part of the blue line was seen introduced						
4	S – Odon			D2	M4	
5	U – Forceps			D1	M2	
6	S – Odon			D2*		
7	U – Forceps	O2	W2	D1	M5	AD
Accidental pressing of the deflation button first noted						
Altered the angle of device insertion						
Application during a maternal contraction introduced, use of fundal pressure removed						
8	S – Odon	O3	W3	D2	M6	AD
Opened the sleeve handles during descent to monitor progression of fetal head first noted						
9	U – Forceps	O4	W4	D1		
10	S – Odon			D2	M7	
11	S – Odon	O5	W5	D1	M8	AD
12	S – Odon	O6	W6	D1	M9	AD
13	S – Odon	O7	W7	D3	M2	
14	U – Failed rotational forceps, emergency Caesarean section			D1	M7	AD
15	U – Forceps			D2	M4	
16	U – Forceps	O8	W8	D2	M10	IBP
17	S – Odon			D1		
18	S – Odon			D3	M11	
19	S – Odon			D4		SST
20	U – Rotational forceps			D4		IBP
21	U – emergency Caesarean section			D3	M6	
22	U – Forceps			D4		
23	U – Forceps			D1	M6	IBP
24	S – Odon			D1		
25	U – Forceps			D4		
26	U – Forceps			D4		
27	S – Odon			D1		AD
28	U – Forceps			D1		
29	S – Odon			D5		
30	U – Forceps			D1		
31	U – Forceps			D4		IBP
32	U – Forceps			D1		
33	S – Odon			D5		
34	S – Odon			D2		
35	S – Odon			D2		
36	U – Forceps			D4		
Odon summit held						
37	U – Forceps			D1		
38	S – Odon			D4		
39	S – Odon			D4	M6	
40	U – Forceps			D3		

*Qualitative interview from Obstetrician not obtained for this birth
O = observation, W = woman, D = Obstetrician, M = Midwife
AD = accidental deflation, IBP = ineffective bulb pump, SST = significant sleeve tear
Bold italic steps = key stages in the study that impacted on technique

For peer review only - http://bmjopen.bmj.com/site/about/guidelines.xhtml

For peer review only

For peer review only - http://bmjopen.bmj.com/site/about/guidelines.xhtml

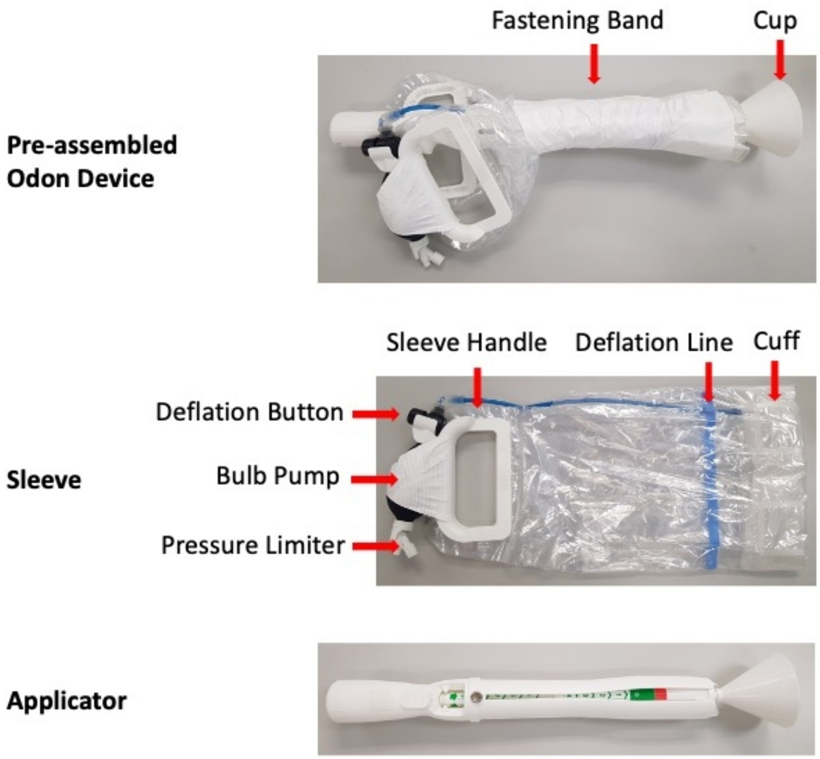

Diagram of the Odon Device

225x190mm (81 x 81 DPI)

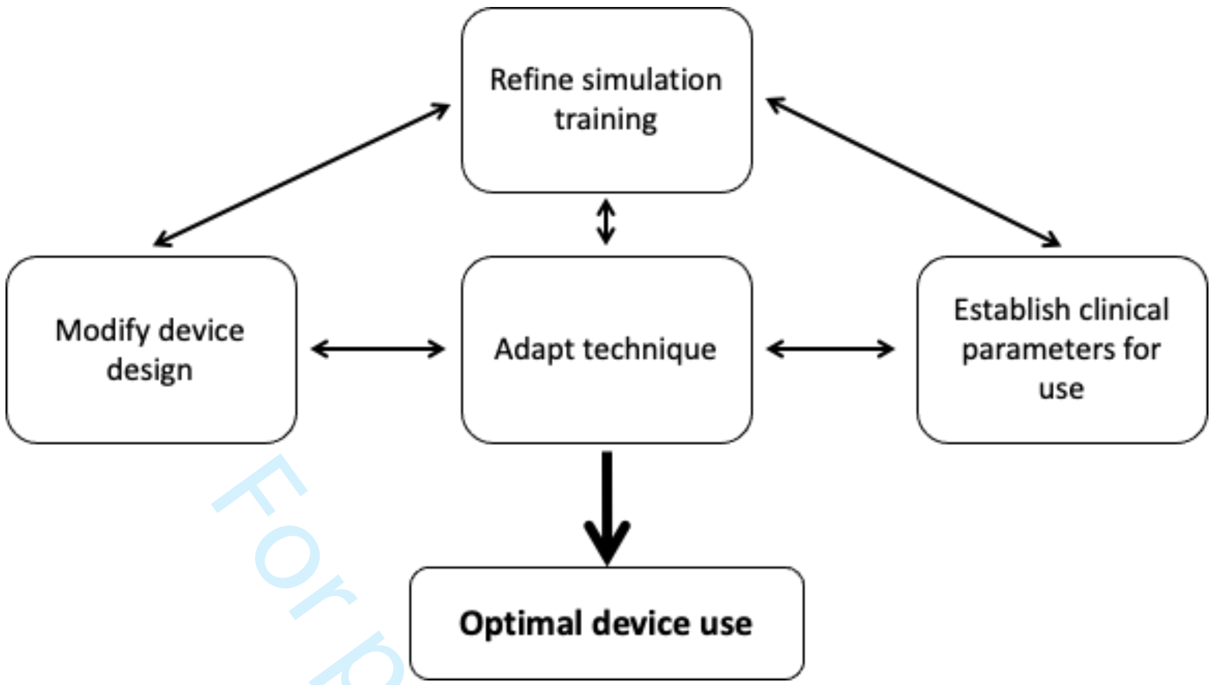

For peer review only

REVISION RECORD		
REV (VER)	REVISION DESCRIPTION	CR NO.
01(A)	INITIAL RELEASE	CM500000111539

BD Odon Device™

Inflatable device for assisted vaginal birth – Instructions For Use

Device Description

The **BD Odon Device™** is an instrument for assisted vaginal birth. The device uses a polyethylene sleeve and an inflatable air cuff to help ease the fetal head out of the birth canal. The BD Odon Device consists of an applicator, sleeve and fastening band.

Indications for Use

The BD Odon Device is envisaged to perform assisted vaginal birth. The main indications for the BD Odon Device are as follows:

- Prolonged second stage of labour
- Suspected fetal compromise in the second stage of labour
- Other medical indications to shorten second stage of labour

Contraindications

The use of the BD Odon Device is contraindicated in the following conditions:

- Cervix is not fully dilated
- Cephalopelvic disproportion/ irreducible moulding and large caput indicative of cephalopelvic disproportion
- Fetal head not fully engaged
- Undefined presentation
- Breech, transverse, face, brow presentations
- Membranes are not ruptured
- Contraindications for vaginal deliveries
- Untrained operator

General Warnings

1. The BD Odon Device should be used by skilled birth attendants who are:
   a. Trained to recognize the conditions for safe and effective application of the BD Odon Device:
      - Full cervical dilation
      - Fetal head position defined
      - Fetal head station defined

   b. Trained to use the BD Odon Device

2. The BD Odon Device is intended for single use. Do not reuse. Device function may be impaired through reuse or cleaning. Degradation of materials or failure to correctly reassemble the device could result in harm for the mother and the fetus. Reusing the product after exposure to biological materials may cause adverse patient reactions due to contamination.

3. As with other devices for assisted vaginal birth, the BD Odon Device could fail to deliver the baby.
   The criteria to define failure and to abandon the procedure are:

   a. No progression in descent of the fetal head after a total of three pulls
   b. No more than two attempts at application (no more than two device slippages)
   c. Duration of procedure longer than 20 minutes

4. As with other instruments for assisted vaginal birth, use of the BD Odon Device may result in maternal and neonatal complications. The most common complications with the use of forceps and ventouse include maternal trauma, neonatal facial injury (forceps), cephalohaematoma, subgaleal haemorrhage, and retinal haemorrhage (ventouse).
   Presently human, animal and simulated studies have not identified potential complications specific to the BD Odon Device therefore this section will be updated after analyses of the safety outcomes of the proposed randomised clinical study.

5. This BD Odon Device is exclusively for clinical investigation only.

6. Ensure packaging is not damaged prior to use. Discard product if packaging is found to be damaged.

General Precautions

As with other devices for assisted vaginal birth the following precautions should be taken before applying the BD Odon Device to ensuring that conditions for safe application of device are met:

- Verify full dilation of cervix, fetal head station at the ischial spines or below
- Verify cephalic vertex presentation (Occiput anterior, Occiput posterior, Occiput transverse positions)
- Verify rupture of membranes
- Provide adequate analgesia according to facility procedures
- Position women in lithotomy position
- Empty bladder
- Re-confirm fetal position
- Lubricate the birth canal

Symbol Legends

Symbol	Meaning
STERILE R	Sterilized using radiation
	Do not re-use
	Do not re-sterilize
	Do not use if package is damaged
	Fragile, handle with care
	Keep away from sunlight
	Keep dry
LATEX	Contains or presence of natural rubber latex
LOT	Batch code
	Use-by date
	Date of manufacture
	Manufacturer
EC REP	Authorized representative in the European Community
i	Consult instructions for use
	Caution
SN	Serial number

Manufacturer: Becton Dickinson Medical Products Pte Ltd, 30 Tuas Avenue 2, Singapore 639461

EC REP Authorised representative in the European Community:
Becton Dickinson France SAS, 11 rue Aristide Bergès, ZI des Iles, 38800 Le Pont de Claix, France

Odon Device is a trademark of Air Bag One. BD, the BD Logo, and all other trademarks are property of Becton, Dickinson and Company. © 2017 BD

For peer review only

VENDOR INFO:

-HOLDING LINES ONLY... DO NOT PRINT.
NOT USE FOR DIMENSIONAL PURPOSES
-IFU DIMENSIONS TO BE 210MM X 297MM.

PMS2755

BD

BDMP R&D
30 TUAS AVENUE 2 S(639461)
SINGAPORE

THIS MATERIAL IS THE CONFIDENTIAL AND PROPRIETARY PROPERTY OF BECTON DICKINSON AND COMPANY. NO PORTION MAY BE USED, REPRODUCED OR DISTRIBUTED WITHOUT PRIOR WRITTEN PERMISSION.

REV (VER) 01(A)	TITLE BD Odon Device™ - Instruction For Use			
CR NO. CM500000111539	DESIGNED BY Narayanan V	UNITS METRIC/INCHES UNLESS OTHERWISE SPECIFIED	SHEET 1 OF 2	TYPE EXPERIMENTAL
REVISION DATE -	DRAFTED BY Narayanan V	SCALE Scale Size 1:1 NOT PRINTED TO SCALE		DRAWING NUMBER SRD-DGP0036
	CREATION DATE 24 NOV 2017			
	FILE NAME SRD-DGP0036_rev 01			

Document: SRD-DGP0036
Valid From: 07-Dec-2017 To: 31-Dec-9999
Print Date: 07-Dec-2017 05:24:29 GMT Standard Time

Doc Type: ZLB
Doc Part: EN
Usage: Prototype Usage

Status: Released EFFECTIVE
Revision: 01 Change #: 500000111539
Version: A Classification: Restricted

DEVICE USE STEPS (FOR CLINICAL INVESTIGATION ONLY)

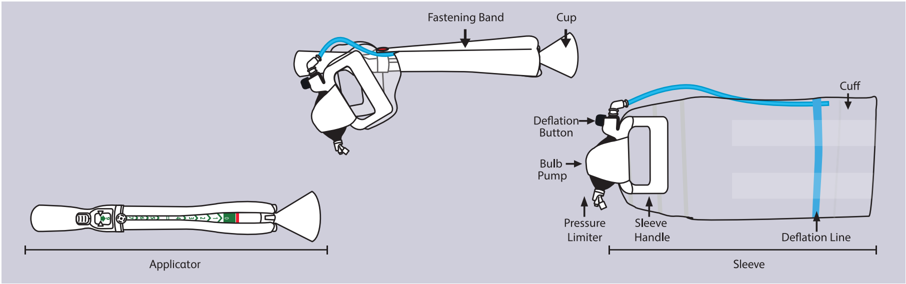

1. Ensure conditions for safe application of device are met:
   a. Full dilation of cervix, fetal head 0 to + 3 station, cephalic vertex presentation (OA, OP, OT positions), rupture of membranes
   b. Provide adequate analgesia according to facility procedures
   c. Position women in lithotomy position
   d. Empty bladder
   e. Re-confirm fetal position
   f. Lubricate the birth canal

2. Remove BD Odon Device from packaging without compromising the sterility of the device.

3. Pull back the fastening band until the blue deflation line is exposed.

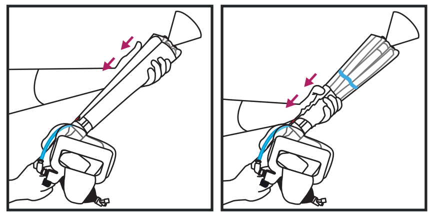

4. With the fastening band still intact, generously lubricate the inside and outside of the sleeve and the cup.

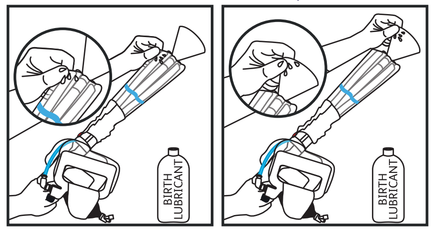

5. While holding the applicator handle gently slide the fastening band back to the top of the sleeve.

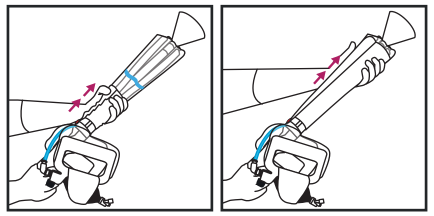

6. Grip the applicator handle and ensure the viewing window is facing upwards.

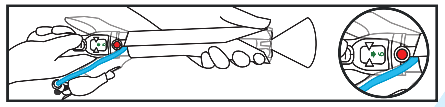

7. Fold the cup and gently insert it through the vulva and check it has regained its circular shape.

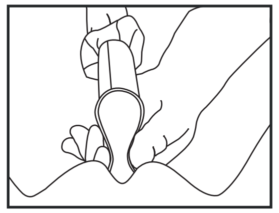

8. Check that there is no maternal tissue trapped between the cup and the fetal head.

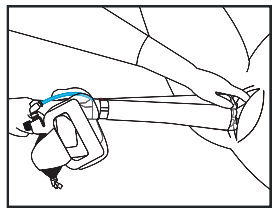

9. With your hand on the handle of the applicator, gently push the device through the vulva. Use your other hand to guide the device and do not apply any force with it.

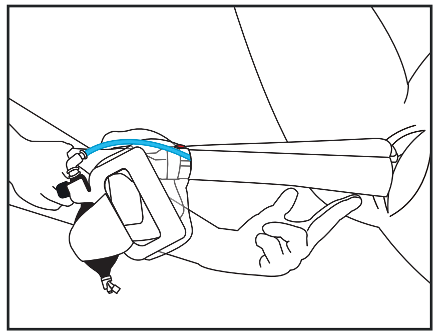

A

DEVICE USE STEPS (FOR CLINICAL INVESTIGATION ONLY)

10. Unfasten the red button

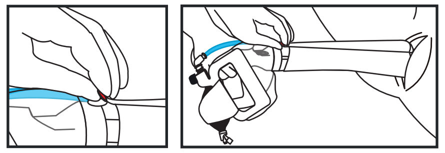

11. Open and completely remove the fastening band.

Note: ensure the sleeve and applicator remain in place inside the vulva.

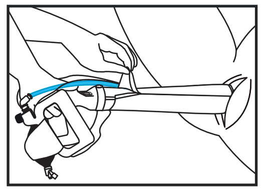

12. Between contractions, keeping both hands away from the sleeve, continue to gently push the applicator, starting at 45° below the horizontal and following the curvature of the birth canal. Monitor progress by looking through the viewing window.

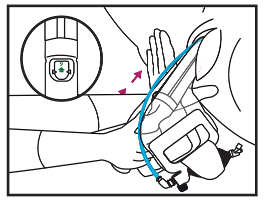

13. Continue to insert the device and stop when "0" appears in the viewing window.

Note: device is fully inserted when "0" appears in the viewing window.

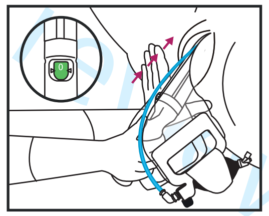

14. Squeeze the bulb pump fully and firmly at least 8 times to inflate the cuff.

Note: There is a pressure limiter in the bulb which prevents over inflation of the cuff.

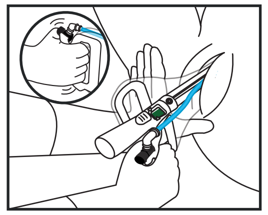

15. While protecting the perineum with one hand, use the other hand to completely withdraw the applicator and cup, leaving only the sleeve in place.

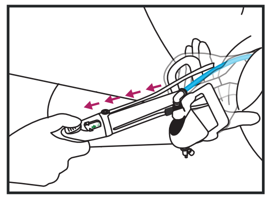

16. To compensate for possible reduction in cuff pressure, squeeze the bulb pump fully and firmly 2 more times prior to traction.

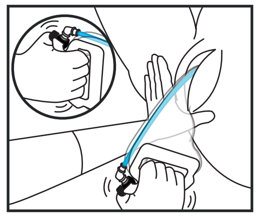

17. Grasp the sleeve handle, and during contractions pull gently and progressively following the J-shape of the birth canal.

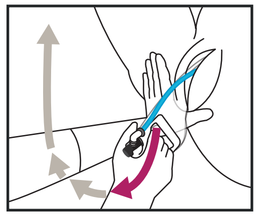

18. While continuing to pull gently along the J-shape of the birth canal, confirm the fetal head is descending with pulling efforts.

Note: if baby is not delivered at the first contraction repeat steps 16, 17 and 18 with any subsequent contractions.

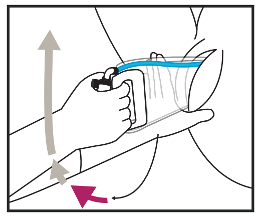

19. Once you see the blue deflation line completely (approximately at crowning) deflate the cuff by pushing on the blue deflation button. Pull the sleeve handle continuing to press the deflation button following the J-shape of the birth canal.

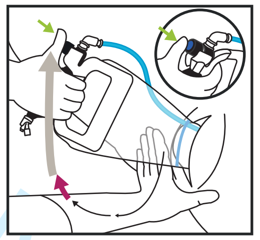

20. Continue to pull the sleeve handle while pressing the blue deflation button to pull the fetal head until the sleeve detaches from the head.

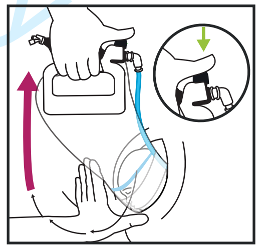

21. Proceed to assist the birth of the baby as per normal procedure.

22. Discard disposable applicator and sleeve according to local appropriate procedure. Do not reuse.

B

BD

BDMP R&D
30 TUAS AVENUE 2 S(639461)
SINGAPORE

THIS MATERIAL IS THE CONFIDENTIAL AND PROPRIETARY PROPERTY OF BECTON DICKINSON AND COMPANY. NO PORTION MAY BE USED, REPRODUCED OR DISTRIBUTED WITHOUT PRIOR WRITTEN PERMISSION.

REV (VER) 01(A)	TITLE BD Odon Device™ - Instruction For Use			
CR NO. CM500000111539	DESIGNED BY Narayanan V	UNITS METRIC/INCHES UNLESS OTHERWISE SPECIFIED	SHEET 2	TYPE EXPERIMENTAL
REVISION DATE -	DRAFTED BY Narayanan V	SCALE Scale Size 1:1	OF 2	DRAWING NUMBER SRD-DGP0036
	CREATION DATE 24 NOV 2017	NOT PRINTED TO SCALE		
	FILE NAME SRD-DGP0036_rev 01			

VENDOR INFO:

-HOLDING LINES ONLY... DO NOT PRINT.
NOT USE FOR DIMENSIONAL PURPOSES
-IFU DIMENSIONS TO BE 210MM X 297MM.

PMS2755

For peer review only

Document: SRD-DGP0036 Doc Type: ZLB Status: Released EFFECTIVE
Valid From: 07-Dec-2017 To: 31-Dec-9999 Doc Part: EN Revision: 01 Change #: 500000111539
Print Date: 07-Dec-2017 05:24:29 GMT Standard Time Usage: Prototype Usage Version: A Classification: Restricted

Table S3 Standards for Reporting Qualitative Research (SRQR)

No.	Topic	Page number in manuscript
	**Title and abstract**	
S1	Title	1
S2	Abstract	2-3
	**Introduction**	
S3	Problem formulation	5-8
S4	Purpose or research question	5
	**Methods**	
S5	Qualitative approach and research paradigm	5-6
S6	Research characteristics and reflexivity	5-7
S7	Context	5-6
S8	Sampling strategy	6
S9	Ethical issues pertaining to human subjects	18
S10	Data collection methods	7
S11	Data collection instruments and technologies	7
S12	Units of study	7
S13	Data processing	7-8
S14	Data analysis	7-8
S15	Techniques to enhance trustworthiness	6-8
	**Results/findings**	
S16	Synthesis and interpretation	8-13
S17	Links to empirical data	8-13
	**Discussion**	
S18	Integration with prior work, implications, transferability, and contribution(s) to the field	14-17
S19	Limitations	14-17
	**Other**	
S20	Conflicts of interest	17
S21	Funding	8

For peer review only

No.	Topic	Item
	**Title and abstract**	
S1	Title	Concise description of the nature and topic of the study Identifying the study as qualitative or indicating the approach (e.g., ethnography, grounded theory) or data collection methods (e.g., interview, focus group) is recommended
S2	Abstract	Summary of key elements of the study using the abstract format of the intended publication; typically includes background, purpose, methods, results, and conclusions
	**Introduction**	
S3	Problem formulation	Description and significance of the problem/phenomenon studied; review of relevant theory and empirical work; problem statement
S4	Purpose or research question	Purpose of the study and specific objectives or questions
	**Methods**	
S5	Qualitative approach and research paradigm	Qualitative approach (e.g., ethnography, grounded theory, case study, phenomenology, narrative research) and guiding theory if appropriate; identifying the research paradigm (e.g., postpositivist, constructivist/ interpretivist) is also recommended; rationale[b]
S6	Researcher characteristics and reflexivity	Researchers' characteristics that may influence the research, including personal attributes, qualifications/experience, relationship with participants, assumptions, and/or presuppositions; potential or actual interaction between researchers' characteristics and the research questions, approach, methods, results, and/or transferability
S7	Context	Setting/site and salient contextual factors; rationale[b]
S8	Sampling strategy	How and why research participants, documents, or events were selected; criteria for deciding when no further sampling was necessary (e.g., sampling saturation); rationale[b]
S9	Ethical issues pertaining to human subjects	Documentation of approval by an appropriate ethics review board and participant consent, or explanation for lack thereof; other confidentiality and data security issues
S10	Data collection methods	Types of data collected; details of data collection procedures including (as appropriate) start and stop dates of data collection and analysis, iterative process, triangulation of sources/methods, and modification of procedures in response to evolving study findings; rationale[b]
S11	Data collection instruments and technologies	Description of instruments (e.g., interview guides, questionnaires) and devices (e.g., audio recorders) used for data collection; if/how the instrument(s) changed over the course of the study
S12	Units of study	Number and relevant characteristics of participants, documents, or events included in the study; level of participation (could be reported in results)
S13	Data processing	Methods for processing data prior to and during analysis, including transcription, data entry, data management and security, verification of data integrity, data coding, and anonymization/deidentification of excerpts
S14	Data analysis	Process by which inferences, themes, etc., were identified and developed, including the researchers involved in data analysis; usually references a specific paradigm or approach; rationale [b]
S15	Techniques to enhance trustworthiness	Techniques to enhance trustworthiness and credibility of data analysis (e.g., member checking, audit trail, triangulation); rationale[b]
	**Results/findings**	
S16	Synthesis and interpretation	Main findings (e.g., interpretations, inferences, and themes); might include development of a theory or model, or integration with prior research or theory
S17	Links to empirical data	Evidence (e.g., quotes, field notes, text excerpts, photographs) to substantiate analytic findings
	**Discussion**	
S18	Integration with prior work, implications, transferability, and contribution(s) to the field	Short summary of main findings; explanation of how findings and conclusions connect to, support, elaborate on, or challenge conclusions of earlier scholarship; discussion of scope of application/ generalizability; identification of unique contribution(s) to scholarship in a discipline or field
S19	Limitations	Trustworthiness and limitations of findings
	**Other**	
S20	Conflicts of interest	Potential sources of influence or perceived influence on study conduct and conclusions; how these were managed
S21	Funding	Sources of funding and other support; role of funders in data collection, interpretation, and reporting

[a]The authors created the SRQR by searching the literature to identify guidelines, reporting standards, and critical appraisal criteria for qualitative research; reviewing the reference lists of retrieved sources; and contacting experts to gain feedback. The SRQR aims to improve the transparency of all aspects of qualitative research by providing clear standards for reporting qualitative research.

[b]The rationale should briefly discuss the justification for choosing that theory, approach, method, or technique rather than other options available, the assumptions and limitations implicit in those choices, and how those choices influence study conclusions and transferability. As appropriate, the rationale for several items might be discussed together.

ACADEMIC MEDICINE

only
